# Intensified electrochemiluminescence and photoluminescence *via* supramolecular anion recognition interactions†

**DOI:** 10.1039/d4sc03338h

**Published:** 2024-07-04

**Authors:** Jun Cheng, Liuqing Yang, Ruiyao Wang, James A. Wisner, Zhifeng Ding, Hong-Bo Wang

**Affiliations:** a Key Laboratory of Optoelectronic Chemical Materials and Devices, Ministry of Education, School of Optoelectronic Materials and Technology, Jianghan University Wuhan Hubei 430056 China hongbo.wang@jhun.edu.cn; b Department of Chemistry and Centre for Advanced Materials and Biomaterials, The University of Western Ontario 1151 Richmond Street London Ontario N6A 5B7 Canada zfding@uwo.ca; c XJTLU Wisdom Lake Academy of Pharmacy, Xi'an Jiaotong-Liverpool University 111 Ren'an Road Suzhou Jiangsu 215123 China; d Department of Chemistry, University of Liverpool Crown Street Liverpool L69 7ZD UK

## Abstract

Herein, intensified electrochemiluminescence (ECL) and photoluminescence (PL) *via* supramolecular anion recognition interactions are demonstrated. A bisindolylpyrrole derivative with a structure containing two indole groups and 2-hexyl-pyrrolo[3,4-*c*]pyrrole-1,3(2*H*,5*H*)-dione, BIPPD, was designed and synthesized *de novo* to induce the enhanced ECL and PL emission based on hydrogen bonding interactions with the dihydrogen phosphate anion. Remarkably, the ECL quantum efficiency and PL quantum yield were discovered to increase up to 5.5-fold and 1.5-fold, respectively, *via* this anion coordination. Dopant PF_6_^−^ was found not to form hydrogen bonds, while HSO_4_^−^ doping does slightly with the receptor molecule. There was no enhancement in either ECL or PL in both scenarios, revealing great recognition selectivity of the synthesized BIPPD. Mechanistic studies *via*^1^H NMR, ECL, and PL spectra illustrated that the ECL processes varied in the presence and absence of H_2_PO_4_^−^ doping, thus leading to the understanding of enhanced efficiency. The bisindolylpyrrole derivative will find applications in supramolecular and analytical chemistry *via* controlled hydrogen bonding interactions.

## Abstract 2

Herein, intensified electrochemiluminescence (ECL) and photoluminescence (PL) *via* supramolecular anion recognition interactions are demonstrated. This great improvement is attributed to the restriction of intramolecular rotation upon anion coordination.

## Graphical abstract

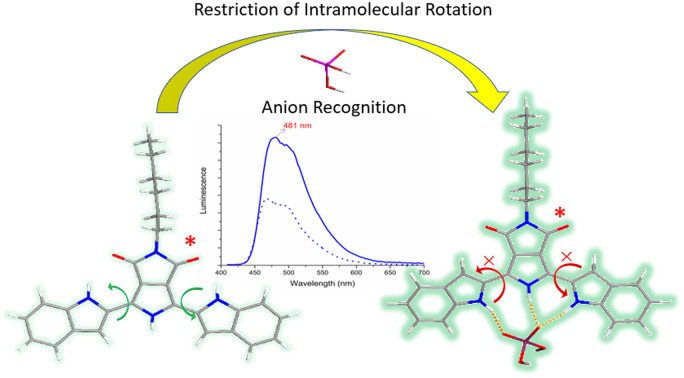

## Introduction

Anions are very relevant to biology, health, and environmental events; thus, anion chemistry has seen great developments from selective recognition to applications in sensing, catalysis, membrane transportation, and responsive materials, and uses as templates for supramolecular architectures.^1–8^ Anion coordination induces the appearance or removal of energy levels between the highest occupied molecular orbital (HOMO) and lowest unoccupied molecular orbital (LUMO) of the anion receptor.^9–12^ This coordination can result in enhanced fluorescence emission. Moreover, recognition interactions of the anion and its receptor can improve the overall rigidity of the complex, thus making non-radiative energy loss from the excited states less probable. In turn, the fluorescence intensity has been reported to increase.^13^ There are some examples regarding anion-complexation-induced enhancement of fluorescence emission. Wu and co-workers reported that a tetrakis(bisurea)-decorated tetraphenylethene (TPE) ligand displays a large fluorescence enhancement in the presence of phosphate ions because of the restricted intramolecular rotation (RIR) of TPE by anion coordination.^14^ Fluoride anions could induce the self-assembly of a tripodal naphthalimide functionalized trimesic amide derivative into a supramolecular polymer, thus leading to strong aggregation-induced enhanced luminescence emission.^15^ Sessler and Gong *et al.* demonstrated that anion-mediated disaggregation of the excimer formed from a cationic macrocycle leads to an increase in the observed fluorescence intensity.^16^ Haley and Johnson reported a series of bis(arylethynyl)pyridine derivatives showing aggregated fluorescence response, which could be used as selective turn-on anion sensors.^17,18^

Electrochemiluminescence (ECL),^19–46^ also known as electrogenerated chemiluminescence, is a light-emission process based on the charge transfer reaction between radical species electrogenerated from a luminophore. Compared with photoluminescence (PL), ECL has higher sensitivity and lower detection limits due to the nonrequirement of a light source and thus has been extensively used for sensing, bioanalysis and clinical diagnostics.^37,38,47,48^ Furthermore, organic light-emitting devices based on ECL gels with an electrolyte, ECL luminophore, and network matrix are complementary platforms to conventional electroluminescent devices.^39,40^

RIR of luminophores upon aggregation/crystallization can prevent excited state energy loss and is expected to improve ECL efficiency.^49–66^ Xu and co-workers studied the ECL of carboranyl carbazoles in aqueous media, which has much higher ECL stability and intensity in the aggregation state. The Yuan^50^ and Lu^51^ groups observed a very strong ECL in hexagonal tetraphenylethylene microcrystals and 1,1-disubstituted 2,3,4,5-tetraphenylsiloles in aqueous solution, respectively, which could be well explained by RIR. Our research groups also discovered a newly synthesized di-boron complex (DBC) with both PL and ECL enhanced when in the crystalline lattice relative to the DBC solution, which was attributed to RIR.^52^ The immobilization of 9,10-anthracene dibenzoate (adb) in porous ultra-thin Zr_12_-adb nanoplates led to excellent ECL performance by means of RIR to suppress unnecessary energy losses due to self-rotation.^53^ Thus, improving the ECL efficiency by restricting intramolecular motions (RIM) is very desirable. Anion coordination may form a rigid complex and further lead to PL enhancement. Indeed, there are some reported anion-induced photoluminescent materials.^3,6,7,11^ Anion recognition is expected as an alternative strategy to RIR/RIM and thus improves ECL efficiency.

**Fig. 1:**
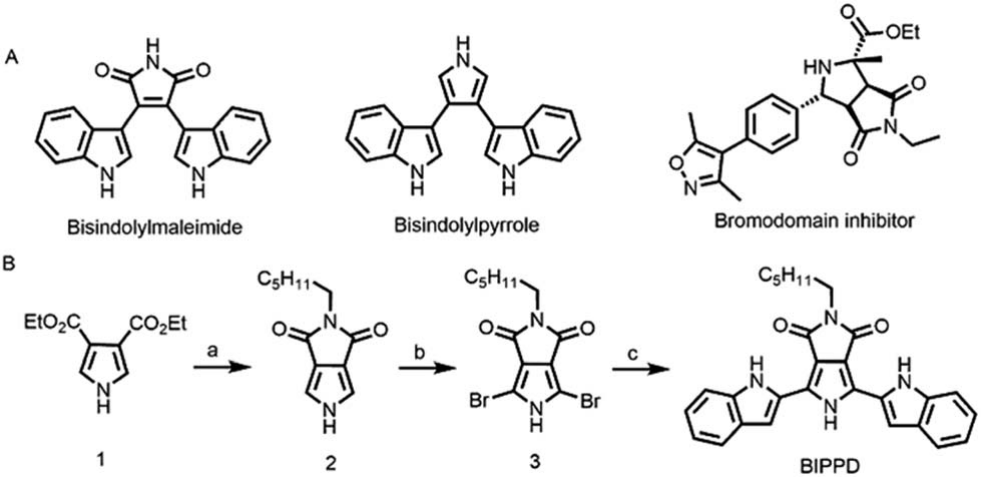
(A) Structures of some pyrrole derivatives (bisindolylmaleimide, bisindolylpyrrole and the bromodomain inhibitor containing pyrrolo[3,4-*c*]pyrrole); and (B) synthetic route to the 2-hexyl-4,6-bis(1*H*-indole)pyrrole[3,4-*c*]pyrrole-1,3-(2*H*,5*H*)-dione, BIPPD. (a) (i) NaOH; (ii) diluted HCl solution; (iii) DCC, hexylamine, THF; (iv) SOCl_2_, DMF. (b) NBS, DMF. (c) Pd(PPh_3_)_2_Cl_2_,K_2_CO_3_, DMF : H_2_O = 5 : 1 (v/v).

Pyrrole derivatives in Fig. 1A are important heterocyclic compounds that are found in numerous functional materials and natural products including in biological probes and chemical sensors. For example, pyrroles and indoles have been extensively used as anion receptors in the field of supramolecular chemistry.^67,68^ Bisindolylmaleimides have both valuable pharmacological properties and potential applications as light-emitting materials.^69–72^ The pyrrolo[3,4-*c*]pyrrole motif is frequently found in numerous bioactive pharmaceutical ingredients.^73^ In addition, pyrrolo[3,4-*c*]pyrroles have been used for the construction of high-performance small molecules and semiconducting polymers for organic thin film transistors and organic photovoltaics.^74–76^ Here, we report the photoelectrochemistry and photochemistry of a specially designed bisindolylpyrrole compound, 2-hexyl-4,6-bis(1*H*-indole)pyrrole[3,4-*c*]pyrrole-1,3-(2*H*,5*H*)-dione (BIPPD) that illustrates how anions can be utilized to restrict molecular motion and further enhance both ECL and PL through strong hydrogen bonding interactions upon addition of the dihydrogen phosphate anion (H_2_PO_4_^−^) anion, Fig. 1B.

## Results and discussion

### Molecule design and synthesis

Phosphate recognition by synthetic anion receptors has flourished in recent years due to its important roles in many biological and industrial applications.^77^ The large size of anionic phosphates makes them less ideal point charges which diminish their extent of electrostatic binding efficiency. As such, designing anion receptors that can recognize and bind phosphates is challenging but desirable for synthetic and supramolecular chemists. A molecule with a “dipterous” structure (like a dragonfly with two wings) has been designed to achieve the aim of RIR/RIM by hydrogen bonding. The “body” and “wings” are composed of a pyrrole derivative and two indole structures, respectively. Both of them are excellent anion receptors due to the existence of NH groups. According to reported literature data, the p*K*_a_ values of pyrrole (23.0) and indole (20.9) were measured in DMSO.^78^ Considering that BIPPD can be approximated as composed of two indole molecules and one pyrrole molecule, the p*K*_a_ value of BIPPD in DMSO should be between 20.9 and 23.0. There is expected to be a strong hydrogen bond interaction in chlorobenzene due to the solvent's non-competitive nature regarding hydrogen bonding.^79^Fig. 1B shows the synthetic route of the target compound BIPPD. The “body” was prepared according to the literature reported by Pollack *et al.* and Sessler's group^80,81^ and the wings were built up by a conventional Suzuki–Miyaura coupling reaction with *N*-Boc-indole-2-boronic acid as a reactant using a palladium catalyst to produce the “dipterous” compound BIPPD. ^1^H NMR and ^13^C NMR characterization results are in the ESI, Fig. S1–S7.†

### ECL enhancement of the BIPPD anionic complex (BIPPDAC)

**Fig. 2:**
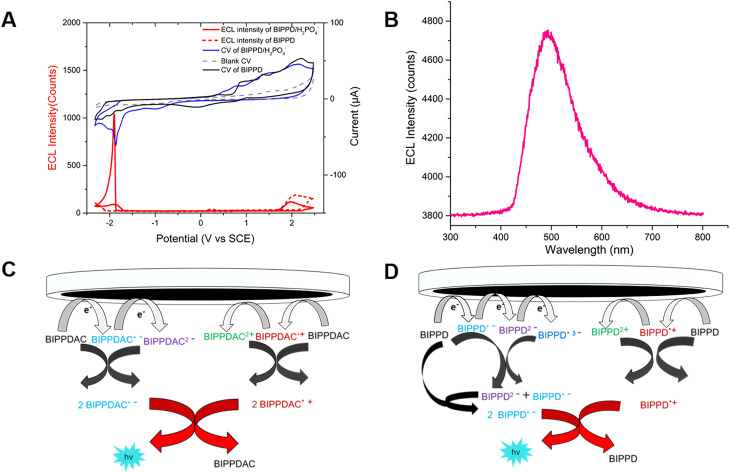
(A) CV and the corresponding ECL voltage curve of 1 mM BIPPD doped with seven equivalents of H_2_PO_4_^−^ in anhydrous acetonitrile with 0.1 M TBAP as the supporting electrolyte at a scan rate of 0.1 V s^−1^; (B) the ECL emission spectrum of BIPPD/H_2_PO_4_^−^ in the annihilation mechanism obtained in a 300 s pulsing experiment. (C) Proposed ECL mechanism of BIPPDAC; and (D) proposed ECL mechanism of the BIPPD anionic complex.

Cyclic voltammetry (CV) measurements in acetonitrile solution containing 0.1 M tetra-*n*-butylammonium hexafluorophosphate (TBAPF_6_) were performed to investigate the ECL and electrochemical performance of both undoped and doped BIPPD with a potential window between −2.30 and 2.50 V *vs.* SCE at a scan rate of 0.1 V s^−1^. The corresponding ECL–voltage curve of BIPPD doped with seven equivalents of H_2_PO_4_^−^ shows higher cathodic ECL peaks of 1039 counts (red curve in Fig. 2A) than that of undoped BIPPD of 101 counts (red dashed curve in Fig. 2A). The ECL efficiency relative to Ru(bpy)_3_^2+^ was improved 5.5-fold from 0.2% to 1.1%. On the contrary, there is not much enhancement in ECL intensity in the anodic region. These different results for ECL in the cathodic and anodic regions reveal that the radical anion of BIPPD doped with H_2_PO_4_^−^ is less stable than its radical cation. The low cathodic and anodic ECL peaks (red dashed curve in Fig. 2A) are attributed to the short lifetimes of unstable BIPPD radicals. In view of the three amine groups and the H_2_PO_4_^−^ anion (good hydrogen bonding donors and acceptor, respectively), this ECL enhancement could be attributed to hydrogen bonding interactions between BIPPD and H_2_PO_4_^−^ that rigidifies the molecule with RIR/RIM and reduces the energy loss in non-radiative indole rotations of the excited state generated *via* annihilation of the electrogenerated radical cation and anion.

Further exploring the details of ECL enhancement, its process was analyzed and mechanisms were proposed (Fig. 2C and D). When the potential was scanned negatively between −2.30 and 2.50 V (blue CV curve, Fig. 2A) for BIPPDAC, two reductive peaks (at −1.79 V and −1.85 V) and three oxidative peaks (at 0.79 V, 1.46 V and 2.12 V) of BIPPDAC were observed (blue curve in Fig. 2A). Besides, the ECL peaks of BIPPDAC in the cathode and anode were noticed at −1.88 V and 1.94 V (red curve in Fig. 2A). This means that the BIPPDAC produces ECL without the involvement of BIPPDAC^3+^. As a result, BIPPDAC was reduced twice to produce dianion BIPPDAC^2−^ in the nearby cathode. The dianion would then react with a neutral BIPPDAC to obtain two radical anions (BIPPDAC˙^−^). In the anodic region, the BIPPDAC loses one electron and produces BIPPDAC˙^+^. Ultimately, the BIPPDAC˙^−^ and BIPPDAC˙^**+**^ produced in the vicinity of the working electrode meet and react with a single electron transferred from the HOMO of BIPPDAC˙^−^ to the HOMO of BIPPDAC˙^+^. They then form the excited species BIPPDAC*, which returns to the ground state, leading to ECL (Fig. 2C).

The CV and ECL–voltage curves for BIPPD doped with different equivalents of H_2_PO_4_^−^ are shown in Fig. S8.† The results indicate that the ECL of BIPPD is maximally enhanced when doped with 7 equivalents of H_2_PO_4_^−^anions. The ECL process proceeded mostly with the second reduction reaction. This implies that the ECL process of BIPPD doped with seven equivalents of H_2_PO_4_^−^anions in acetonitrile solution forms a BIPPD dianionic complex (BIPPDAC) based on hydrogen bonding interactions.

In addition, undoped BIPPD underwent three reductive reactions at peak potentials of −1.75 V, −1.99 V, and −2.16 V; in the anodic scan from 0 to 2.5 V, three oxidation waves were observed at 0.89 V, 1.36 V, and 2.18 V, respectively (black CV curve, Fig. 2A). Meanwhile, ECL peaks of BIPPD were observed at −2.29 V and 2.02 V, respectively. These suggest that BIPPD was reduced three times and oxidized two times to produce BIPPD˙^−^, BIPPD^2−^, and BIPPD˙^3−^ in the cathodic region as well as BIPPD˙^+^ and BIPPD^2+^ in the anodic region successively. One electron was then transferred from BIPPD˙^3−^ to BIPPD to produce BIPPD˙^−^ and BIPPD^2−^ that further reacted with BIPPD to generate two equivalents of BIPPD˙^−^ in the diffusion layer. In the anodic region, electrogenerated BIPPD˙^2+^ reacted with BIPPD to produce two equivalents of BIPPD˙^+^ in the vicinity of the electrode.^54^ Finally, the electrogenerated BIPPD˙^−^ and BIPPD˙^+^ meet together to react with a single electron transferred from the BIPPD˙^−^ HOMO to the BIPPD˙^+^ HOMO, thus forming an excited species of BIPPD* that give off luminescence upon returning to the ground state as outlined in Fig. 2D.

The potential pulsing method was utilized to reduce the time interval between generations of radical anions and cations to further enhance ECL. A potential pulsing experiment was carried out for 300 s in 1 mM BIPPD solution doped with seven equivalents of H_2_PO_4_^−^. The applied potential was pulsed between −2.30 and 2.50 V at a frequency of 5 Hz (Fig. S9†). An ECL emission spectrum was acquired during this time period as displayed in Fig. 2B. The peak wavelength was determined to be 490 nm. The annihilation ECL spectrum of BIPPD cannot be collected due to its weak signal.

### Enhanced PL of BIPPD/H_2_PO_4_^−^ in solution and films

**Fig. 3:**
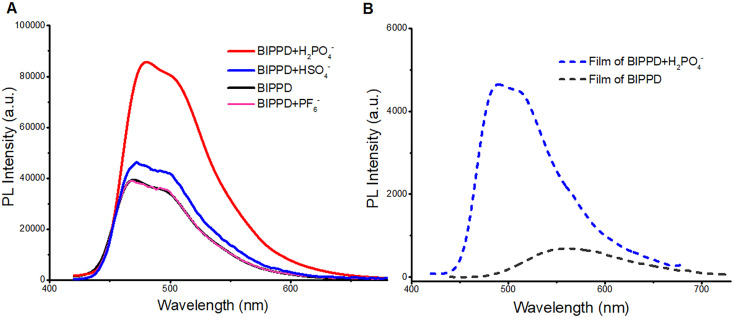
(A) PL spectrum of BIPPD in chlorobenzene (1 × 10^−5^ mol L^−1^, black curve) and BIPPD with one equivalent of H_2_PO_4_^−^ (1 × 10^−5^ mol L^−1^, red curve), HSO_4_^−^ (1 × 10^−5^ mol L^−1^, blue curve) or PF_6_^−^ (1 × 10^−5^ mol L^−1^, pink curve) in chlorobenzene solution excited at 390 nm, respectively; (B) PL spectrum of BIPPD (gray, dashed line) and BIPPD with one equivalent of H_2_PO_4_^−^ (blue, dashed line) in films excited at 390 nm, respectively.

The photophysical properties of BIPPD in chlorobenzene at a concentration of 1 × 10^−5^ mol L^−1^ were investigated by UV-visible absorption and PL spectroscopies. Fig. S10† shows one broad absorption band centered around 360–460 nm (black curve) observed in its UV-visible absorption spectrum. In comparison, the absorption of BIPPD in the presence of H_2_PO_4_^−^ was particularly enhanced at 300–340 nm. This enhancement demonstrates that more excited state molecules are generated from the π–π* transition when BIPPD combines with H_2_PO_4_^−^. This will also lead to the PL enhancement as explained below. Fig. 3A shows the PL spectra of BIPPD with one equivalent of H_2_PO_4_^−^ (red curve), BIPPD with one equivalent of HSO_4_^−^ (blue curve), BIPPD with one equivalent of PF_6_^−^ (pink curve), and BIPPD (black curve) in chlorobenzene. The PL intensity of BIPPD upon the addition of H_2_PO_4_^−^ in chlorobenzene (red curve) is clearly enhanced approximately 1.5-fold relative to the BIPPD solution in the absence of H_2_PO_4_^−^ (black curve). The absolute quantum yield of BIPPD with one equivalent of H_2_PO_4_^−^ is increased from 18.6% to 28.1%. Considering the similar phenomenon of ECL enhancement, it is plausible that there is hydrogen bonding formation between the N–H of BIPPD and the anion of H_2_PO_4_^−^. The shape of the three N–H interaction sites on BIPPD with H_2_PO_4_^−^ is like a clamp that increases greatly RIR of the two indole groups, thus rigidifying the configuration of BIPPD to reduce the energy loss of non-radiative processes of the photoinduced excited states, which in turn increases the PL intensity. A detailed study on PL enhancement by introducing various equivalents of H_2_PO_4_^−^ to the BIPPD solution revealed that the PL intensity was continuously enhanced, as demonstrated in Fig. S11.† It was discovered that the PL intensity increase reached a maximum of two-fold when one equivalent of H_2_PO_4_^−^ was added, and the enhancement remained the same with the addition of more H_2_PO_4_^−^. Actually, ECL and PL respond differently to doping concentration due to differences in their luminescence mechanisms and the processes of charge transport and recombination within the materials. For example, charge transport primarily occurs between the electrode and the electrolyte in ECL and charge transport primarily occurs within the material itself in PL.

The PL of BIPPD upon the addition of one equivalent of either PF_6_^−^ or HSO_4_^−^ was also studied for comparison. The PL intensity remained nearly the same after adding PF_6_^−^ (pink; Fig. 3A) due to the absence of observable hydrogen bonding between PF_6_^−^ and BIPPD under these conditions. There is a tiny improvement in the PL intensity of BIPPD upon the addition of one equivalent of HSO_4_^−^ (blue curve). Considering the components of hydrogen sulfate, it is possible to form extremely weak hydrogen bonding in the BIPPD/HSO_4_^−^ system. The indole groups on both sides of the precursor may still rotate freely, which could cause low luminescence efficiency due to non-radiative processes. Hydrogen bonding interactions are a reliable and controllable way to rigidify this molecule in view of the three amine groups, which are good hydrogen bond donors.

**Fig. 4:**
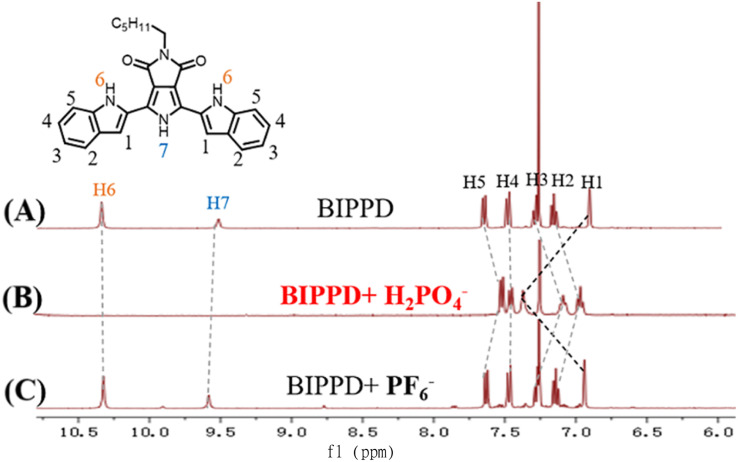
Stacked partially ^1^H NMR spectra of complex (A) BIPPD, (B) BIPPD with one equivalent of H_2_PO_4_^−^, and (C) BIPPD with one equivalent of PF_6_^−^ in CDCl_3_.

Photophysical properties of films of BIPPD and BIPPD/H_2_PO_4_^−^ systems were investigated to further study the PL enhancement of BIPPD. The PL spectrum in Fig. 3B shows that BIPPD in the neat film has a very broad PL emission band with one emission peaks at 560 nm (gray dashed line). The emission was enhanced about nine-fold and blue-shifted about 70 nm after being added with one equivalent of H_2_PO_4_^−^, blue dashed line in Fig. 3B. This blueshift indicates a new conformation of BIPPD after H_2_PO_4_^−^ is added, which is unchanged during the spin-coating process of immobilization to a substrate. The RIR upon aggregation/crystallization during film formation could further prevent excited state energy loss and improve the PL efficiency. The absorption bands of BIPPD (gray) and BIPPD added with the H_2_PO_4_^−^ anion (blue dashed line) in films are similar, and both of them are mainly distributed in the wavelength range between 300 and 500 nm (Fig. S12†). Furthermore, the PL spectrum of the BIPPD film (gray dashed line in Fig. 4B) is red-shifted and broadened relative to that of BIPPD in chlorobenzene (black, Fig. 3A) due to the potential formation of aggregates of BIPPD *via* π–π stacking. The PL spectra of the BIPPD/H_2_PO_4_^−^ system in solutions and in films overlapped well with each other. It is plausible that the conformation of BIPPD mixed with H_2_PO_4_^−^ is the same in solution and the film, and both achieve higher PL emission due to hydrogen bonding interactions.

The ECL, UV-visible absorption, and PL spectra strongly support that the induced ECL and PL emission enhancement of BIPPD added with H_2_PO_4_^−^ is due to hydrogen bonding forming a complex. This complex could rigidify the configuration of BIPPD by hydrogen bonding interactions and limit the non-radiative process. It is evident that the hydrogen bond formation is complementary to CIE^52^ and AIE^82^ discovered by Tang *et al.* in luminescence enhancement.

### ^1^H NMR chemical shift changes upon the addition of anions

The study of phosphate recognition by synthetic anion receptors has flourished in recent years due to its important roles in many biological and industrial applications.^77^ The large size of anionic phosphates makes them less ideal point charges which diminish their extent of electrostatic binding efficiency. As such, designing anion receptors that can recognize and bind phosphates is challenging but very desirable for synthetic and supramolecular chemists. The formation of hydrogen bonds between BIPPD and H_2_PO_4_^−^ was further investigated using ^1^H NMR. The ^1^H NMR spectra were characterized for comparison as shown in Fig. 4. Spectra A and B display the BIPPD in the presence and absence of H_2_PO_4_^−^, respectively. The BIPPD + H_2_PO_4_^−^ system exhibits obvious chemical shift changes upon complexation: the N–H signals of BIPPD (H6 and H7) disappear whilst the two indole C–H signals (H1) move significantly downfield (Δ*δ* = 0.45 ppm). This is similar behaviour to the observations of dihydrogen phosphate anion complexation with other receptors reported.^9,83–85^ Upon the addition of one equivalent of TBAPF_6_, the signals of H1–H7 are nearly identical between spectra A and C, indicating negligible interaction between BIPPD and PF_6_^−^. Examining ^1^H NMR spectra A and B in Fig. 4, it is reasonable to conclude that there are strong interactions between BIPPD and H_2_PO_4_^−^ since they contain good hydrogen bond donors and acceptors.^86–88^ These results support the supposition that the ECL and PL enhancement is due to hydrogen bonds forming between the two species, which restricts intramolecular rotation and rigidifies the configuration of BIPPD to reduce the energy loss of non-radiative processes of the photoinduced excited states. The different enhancement effects of ECL and PL after doping with H_2_PO_4_^−^ are attributed to the varying mechanisms and sensitivities of these two types of luminescence.

### Configuration of BIPPD and the BIPPD + H_2_PO_4_^−^ complex

**Fig. 5:**
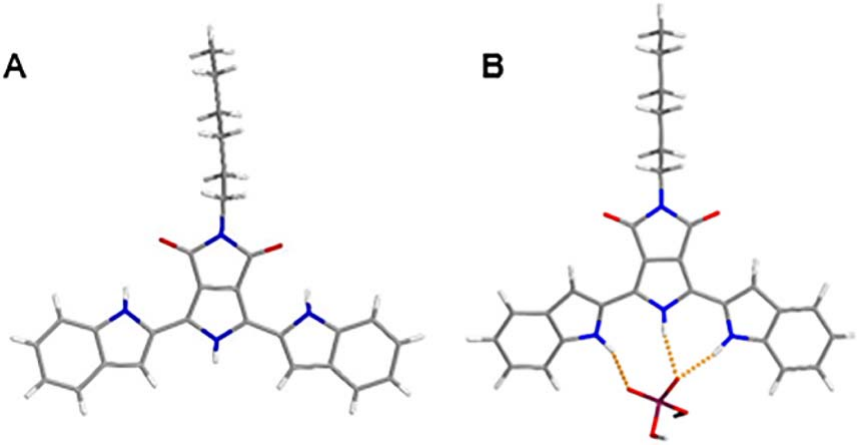
(A) One conformer of BIPPD found within the unit cell in the solid state from the single crystal X-ray structure; (B) energy minimized (HF 6-311+G**) structure of BIPPD + H_2_PO_4_^−^. The indole N̲H⋯H_2_PO̲_4_^−^ distances are 2.882 and 2.795 Å; the pyrrole distance N̲H⋯H_2_PO̲_4_^−^ is 2.865 Å.

To acquire direct evidence for explaining the ECL and PL enhancement, the single crystal of BIPPD was obtained from a BIPPD chloroform solution, Fig. 5A, and extra crystallographic data are demonstrated and listed in ESI, Fig. S13, S14 and Tables S2–S8.† This single crystal of BIPPD displays a coplanar structure in the solid state evident in the front and side views of the determined crystal structure (Fig. 5A and S13†). The two indole groups should rotate freely in solution and luminescence efficiency may be low due to the non-radiative intramolecular motions, as described for a silole by Tang and coworkers^89^ and a di-boron complex by us.^52^ The near coplanarity between the maleimide backbone and the two symmetrical indole groups with the “tail” of the alkyl chain tip-tilted was determined to be 35.2°, Fig. S13.† The “tail” structure can not only greatly improve the solubility of BIPPD in less polar solvents, but also likely prevent close face-to-face intermolecular π–π stacking.

Theoretical geometry minimizations on the receptor BIPPD binding with H_2_PO_4_^−^ were performed (Fig. 5B). The twisted structure of BIPPD is well demonstrated to be rigidified through hydrogen bonding interaction of the pyrrole and two indole NH groups and the anion oxygen acceptors; two oxygen atoms of H_2_PO_4_^−^ would restrict the rotations of two indole moieties to reduce the loss of nonradiative energy. This binding geometry could explain why the ECL and PL of BIPPD were enhanced with the addition of H_2_PO_4_^−^. The spatial arrangement of BIPPD in the presence of H_2_PO_4_^−^ is different from the single crystal structure of BIPPD where the conformations of both the indole groups are flipped by approximately 180° (Fig. 5B) to engage their much stronger NH hydrogen bond donors instead of the CH groups at the 3-positions. This change could also be an interpretation of the 70 nm difference in emission wavelengths of BIPPD in films before and after H_2_PO_4_^−^ is introduced and the NMR observations in the previous section. The association constant *K*_a_ of the BIPPD/H_2_PO_4_^−^ system was determined to be 2.55 × 10^6^ M^−1^ (Fig. S15†) in chlorobenzene based on a UV-visible titration experiment as described in Section S6.† The value is much higher than those of other dihydrogen phosphate complexes reported.^1,2,77^ This indicates tight complexation of BIPPD with H_2_PO_4_^−^ in non-polar solution. Therefore, the film configuration likely remains nearly constant after spin coating. In both cases, the RIR of indole groups by means of hydrogen bond formation with H_2_PO_4_^−^ plays a very important role. The correlating emission wavelengths for the BIPPD/H_2_PO_4_^−^ system in solution and in films also support this conclusion.

### Selectivity recognition for H_2_PO_4_^−^

Based on the above observations, it is plausible that H_2_PO_4_^−^ interacts with BIPPD to form a characteristic co-conformation by hydrogen bonding interactions and enhance greatly BIPPD ECL. ECL probes have been reported for the detection and identification of anions, such as studies by Schmittel's team, which employed ruthenium(ii) and iridium(iii) complexes to detect cyanide and fluoride ions.^90–92^ However, we are unaware of any precedent reported regarding ECL enhancement or quenching *via* supramolecular anion recognition, while a few colorimetric and fluorescent chemosensors for anions including H_2_PO_4_^−^ based on hydrogen bonding interaction^1,2,77^ and only one case on chemiluminescence-based probes for monitoring tyrosinase activity in conjunction with biological thiols^82^ have been reported. The ease of our ECL detection of H_2_PO_4_^−^ and the simplicity of handling described above will make the technique more appealing and applicable to other anion determination.

**Fig. 6:**
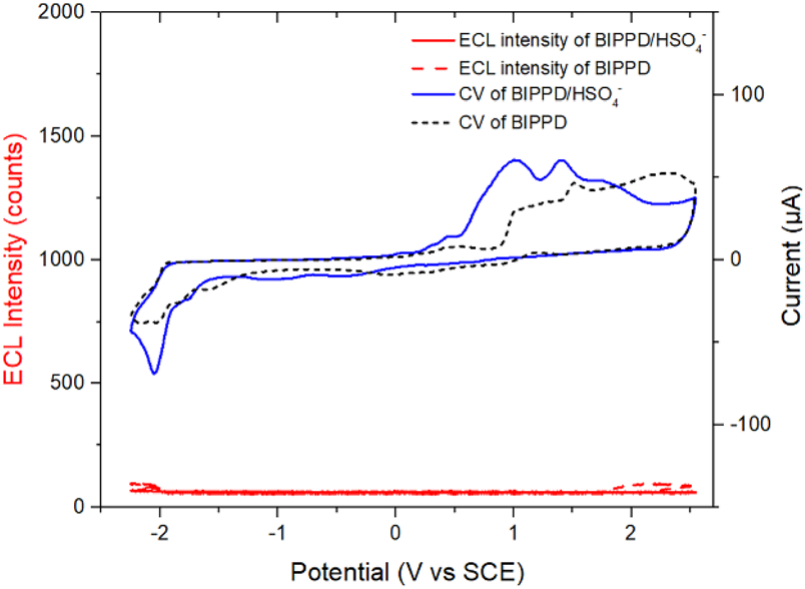
CV and corresponding ECL voltage curves of 1 mM BIPPD doped with seven equivalents of H_2_PO_4_^−^ and HSO_4_^−^ in anhydrous acetonitrile with 0.1 M TBAP as the supporting electrolyte at a scan rate of 0.1 V s^−1^.

To further identify the function of BIPPD in ECL detection, HSO_4_^−^ was selected as another anion to be studied. Upon introduction of HSO_4_^−^, the oxidation peaks were shifted only 0.11 V toward the center potential of 0 V and the reduction peaks were slightly sharpened, as demonstrated in the blue CV curve in Fig. 6, which is similar to the redox behaviors of BIPPD forming hydrogen bonding with H_2_PO_4_^−^. This consistent variation reveals that hydrogen bonding exists between BIPPD and HSO_4_^−^. Nevertheless, there is no ECL enhancement in either the cathodic or anodic region, which means there are limited hydrogen bond interactions between BIPPD and HSO_4_^−^ (red dashed curve in Fig. 6), as reflected in their PL results (blue curve in Fig. 4). This finding is just the reverse of the introduction of H_2_PO_4_^−^ and similar to the PL results. In addition, the PF_6_^−^ does not form appreciable hydrogen bonds with BIPPD when it acts as an electrolyte in this ECL process, which was established by the ^1^H NMR spectral results. Those results demonstrated that the addition of a small amount of H_2_PO_4_^−^ selectively enhances the PL and ECL emission through a combination of hydrogen bond interactions. Therefore, BIPPD is not only a fluorescent chemosensor but also can be an ECL sensor in the selective recognition of H_2_PO_4_^−^.

## Conclusions

In summary, a bisindolylpyrrole derivative BIPPD was designed and successfully synthesized by a traditional Suzuki–Miyaura coupling reaction. For the first time, the ECL was observed to be significantly enhanced *via* H_2_PO_4_^−^ recognition, thus achieving a relative ECL efficiency of 1.1%. The efficiency is 5.5-fold higher than that of undoped BIPPD. Its PL enhancement was observed upon the addition of H_2_PO_4_^−^ anions to form hydrogen bonding between the NH groups of the indole/pyrrole heterocycles and H_2_PO_4_^−^. It is evident that the hydrogen bonding between anion H_2_PO_4_^−^ and receptor molecule BIPPD rigidifies the configuration and restricts the motions of indole groups to reduce the loss of non-radiative energy, thus greatly improving the ECL and PL performance, similar to the mechanism of aggregation-induced emission (AIE) and crystallization-induced emission enhancement (CIEE). While hydrogen bonding exists between BIPPD and HSO_4_^−^, there is no enhancement in ECL intensity. Nevertheless, this mechanism of producing PL and ECL *via* the introduction of anions such as H_2_PO_4_^−^ to form hydrogen bonds is a *de novo* strategy to design and synthesize highly efficient ECL materials for emerging supramolecular and analytical chemistry.

## Experimental

### Materials and apparatus

All reagents and solvents were purchased from commercial suppliers and used without further purification. ^1^H (400 MHz) and ^13^C (100 MHz) NMR spectra were recorded with an Ascend™ 400 MHz spectrometer (Bruker). ^1^H and ^13^C NMR spectra were referenced relative to tetramethyl-silane (TMS) using the residual non-deuterated NMR solvent signal. UV-visible absorption spectra were measured using a UV-1500 spectrophotometer (Macy Instruments Inc., China). Fluorescence spectra were recorded using an FL 8500 Fluorescence Spectrometer (PerkinElmer). Cyclic voltammograms (CVs) and ECL–voltage curves were obtained using an ECL analyzer (HYZ-3002, Xi'an HeYongzhong Electronic Technology Co., Ltd, China) with a photomultiplier tube (PMT) held at 750 V. ECL spectra were collected on an electrogenerated chemiluminescence spectrum system (ECLS-ML, FORTEC Technology (HK), Co., Ltd, China) with a grating of 121 L nm^−1^ blazed at 413 nm and an Andor CCD camera (Model DU401A-BR-DD, UK) cooled to −80 °C.

### Synthesis of BIPPD

Fig. 1B shows the synthetic route of the target compound BIPPD. The compound 3,4-pyrrole dicarboxylic ester 1 was prepared according to the literature reported by Pollack *et al.* and Sessler's group^80,81^ and was then converted into the intermediate 2 with the structure of pyrrolo[3,4-*c*]pyrrole. After bromination, a conventional Suzuki–Miyaura coupling reaction was carried out between intermediate 3 and *N*-Boc-indole-2-boronic acid using a palladium catalyst to produce the target compound BIPPD. More synthetic details are available in the ESI.†

### Photophysical measurements

UV-visible absorption spectra were measured using a UV-1500 spectrophotometer (Macy Instruments Inc., China). Fluorescence spectra were recorded using an FL 8500 Fluorescence Spectrometer (PerkinElmer). UV-visible titration experiments were performed when a chlorobenzene solution of BIPPD (1 × 10^−5^ M) was titrated with a solution of TPA–H_2_PO_4_ from 0 equivalents to 2 equivalents. The data were then uploaded using Excel or CSV to online site http://supramolecular.org. This website was founded in 2015 by Prof. Pall Thordarson and provides free online analysis tools for supramolecular chemistry research including tools for determining binding constants from NMR, UV-visible and fluorescence titration experiments.^93,94^ The association constant K of the BIPPD/H_2_PO_4_^−^ system and associated errors were obtained using these tools.

### ECL measurements and electrochemistry

All electrochemical tests were performed using a three-electrode system including a glassy carbon electrode (GCE) with 3 mm diameter and two Pt wires as the counter electrode (CE) and reference electrode (RE), respectively. The applied potential was calibrated using an internal standard of ferrocene (Fc) for all electrochemical experiments, and the redox potential of Fc^+^/Fc was taken as 0.424 V *versus* the saturated calomel electrode (SCE).^29,95^ Before each test, the glass electrochemical cell was immersed in a base bath (5% KOH isopropanol solution) for 4 hours, and then moved to an acid bath (5% HCl aqueous solution) and immersed for the same time. The GCE was polished with 0.3 micron, 0.2 micron and 0.05 micron alumina slurries successively until a mirror-like surface finish was obtained. The GCE was then sonicated in 0.1 M HNO_3_ solution and ultrapure water and dried with a stream of Ar gas. Further, the polished GCE was scanned in a 1 mM potassium ferricyanide solution containing 0.2 M potassium chloride. When the difference between the cathodic peak and the corresponding anodic peak potentials of [Fe(CN)_6_]^3−^ + e^−^ = [Fe(CN)_6_]^4−^ was less than 70 mV, the GCE was ready for use. The CE and RE were sonicated with acetone, isopropanol, and ultrapure water for 2 min, respectively. Finally, these electrodes were dried at 120 °C in an oven and left to cool to room temperature before the experiment. In ECL studies, a solution containing approximately 1 mM of BIPPD, 0.1 M TBAP as the supporting electrolyte in 5 mL anhydrous acetonitrile (MeCN) was added to the electrochemical cell. To reduce the impact of air, the assembly process was completed in a glove box and the cell was sealed with a custom-made Teflon cap to perform electrochemical and ECL experiments.

### Computational information

Energy minimized geometry optimizations were executed at the HF 6-311+G** level using Spartan '14 from three different initial geometries of the receptor and H_2_PO_4_^−^ beginning with the indole rings in coplanar syn/syn, syn/anti and anti/anti conformations.^96^ The syn/syn binding geometry was the lowest energy of the three models minimized and is the one used here.

## Data availability

All experimental and computational data are summarized in the ESI† and details are available upon request.

## Author contributions

J. C. synthesized all the materials and performed the experiments. H. B. W. conceived the original concept and designed the materials. H. B. W. and Z. D. supervised the project. R. W. did the single crystal X-ray characterization. J. A. W. carried out the computation modelling. J. C., L. Y., Z. D. and H. B. W. discussed the results and contributed to the data interpretation. J. C., Z. D. and H. B. W. wrote the paper. J. C., L. Y., J. A. W., Z. D. and H. B. W. contributed to editing the manuscript. J. C., H. B. W. and Z. D. finalized the paper.

## Conflicts of interest

There are no conflicts to declare.

## Supplementary Material

SC-015-D4SC03338H-s001

SC-015-D4SC03338H-s002
