# Supplementary material for: Intensified electrochemiluminescence and photoluminescence via supramolecular anion recognition interactions
Source: Chem Sci. 2024 Jul 4;15(31):12291–300. doi: 10.1039/d4sc03338h (PMC11304522; doi:10.1039/d4sc03338h)
Supplement: SC-015-D4SC03338H-s001 [file SC-015-D4SC03338H-s001.pdf]

Electronic Supplementary Material (ESI) for Chemical Science.
This journal is © The Royal Society of Chemistry 2024

Electronic Supplementary Information for

Intensified electrochemiluminescence and photoluminescence via supramolecular anion recognition interactions

Jun Cheng, [†a,c,d] Liuqing Yang, [†b] Ruiyao Wang,[c] James A. Wisner,[b] Zhifeng Ding,[*b] and Hong-Bo Wang*[a]

[a] Key Laboratory of Optoelectronic Chemical Materials and Devices, Ministry of Education, School of Optoelectronic Materials and Technology, Jianghan University, Wuhan, Hubei 430056, China. E-mail: hongbo.wang@jhun.edu.cn(H.B.W.)

[b] Department of Chemistry and Centre for Advanced Materials and Biomaterials, The University of Western Ontario, 1151 Richmond Street, London, Ontario N6A 5B7, Canada. E-mail: zfding@uwo.ca (Z.D.)

[c] XJTLU Wisdom Lake Academy of Pharmacy, Xi'an Jiaotong-Liverpool University, 111 Ren'an Road, Suzhou, Jiangsu 215123, China

[d] Department of Chemistry, University of Liverpool, Crown Street, Liverpool L69 7ZD, U.K.

† These authors contributed equally to this work.

Table of Content

1. **Experimental details of synthesis and characterization**
2. **NMR Spectra of all the synthesized compounds**
3. **ECL efficiency calculations**
4. **Absorption and PL spectra**
5. **Crystallographic data of BIPPD**
6. **Equal K/ dimerization**
7. **Theoretical calculation results**

1. Experimental details of synthesis and characterization

3,4-Pyrrole dicarboxylic acid (1): The synthesis procedure of this intermediate can be referred in publications by Pollack *et al.* and the Sessler group. [1-2]

2-hexylpyrrole [3, 4-c]pyrrole-1,3-(2H,5H)-dione (2) : To a suspension of compound **1** (2.75g, 17.7mmol) dissolved in 20 ml tetrahydrofuran (THF), was added a solution of N, N-dicyclohexylcarbodiimine (4.4 g, 21.3mmol) in 30 ml anhydrous THF at nitrogen atmosphere. After stirred and refluxed for 2 hours with a precipitate formed, the mixture was cooled to room temperature and filtered to remove the N, N'-dicyclohexylurea, which could be washed with more THF. The resulting THF solution was concentrated to 15 mL in vacuum, and hexylamine (1 g, 10 mmol) was added via a syringe at N_2 atmosphere. The mixture was subsequently stirred overnight at room temperature. The solvent was removed under vacuum to give a gummy material, and titration with dilute HCl gave a thick paste. An NaOH solution was added and stirred to neutralize the acid and then the solution was filtered to remove excess urea. The filtrate was neutralized with dilute HCl to give a white precipitate, which was filtered and dried giving 1 g 4-(hexylcarbamyl)pyrrole-3-carboxylic acid of white solid (42% yield).

To the solution of compound 4-(hexylcarbamyl)pyrrole-3-carboxylic acid (1 g, 4.18mmol) dissolved in 10 ml of N, N-dimethylformamide (DMF), 15.54 mL of 1mol/L dichloromethane solution of thionyl chloride($SOCl_2$) was added in an ice bath at nitrogen atmosphere. The reaction mixture was stirred at room temperature for 2 hours before quenched by adding a certain amount of ice/water mixture. Saturated NaCl solution was added and extracted 3 times with THF. The organic layer was dried on anhydrous magnesium sulfate, filtered and concentrated. Chromatography of the residue on silica gel using ethyl acetate/petroleum ether (v/v, 1:1) to obtain 0.7g white solid (76%).^{1}H NMR (400 MHz, $CDCl_3$) δ 9.88 (s, 1H), 7.06 (s, 2H), 3.53 (s, 2H), 1.63-1.55 (m, 2H), 1.26 (s, 9H).

2-hexyl pyrrole [3,4-c]2,5-dibromopyrrole 1,3-(2H,5H)-dione (3): Compound **2** (0.7g, 3.2mmol) was dissolved in 20 ml of THF, cooled to -80°C at N_2 atmosphere before N-bromosuccinamide (NBS) (1.2 g,6.7mmol) was added. The temperature was lowered to -70°C for 30 minutes, then to 0°C for 3.5 hours. A small amount of Na_2SO_3 (about 0.1g) was added, stirring for 15 min, then saturated salt water was added to extract with ethyl acetate, and added for dried with anhydrous magnesium sulfate ($MgSO_4$), filtration and concentration. Chromatography of the residue on silica gel using petroleum ether/ethyl acetate (v/v, 4 :) to obtain 0.53g white solid powder (50% yield).^{1}H NMR (400 MHz, $CDCl_3$) δ 9.04 (s, 1H), 3.64-3.46 (m, 2H), 1.59 (s, 5H), 1.29 (d, J = 3.0Hz, 4H), 0.87 (t, J = 6.7Hz, 2H).

2-hexyl-4, 6-bis(1H-indole) pyrrole[3,4-c] pyrrole-1,3-(2H,5H)-dione (BIPPD): In a dry round-bottomed flask, compound **3** (378mg, 1mmol), $PdCl_2(PPh_3)_2$(140mg,20 mol %), N-Boc-indole-2-boronic acid (627mg ,2.4mmol) and potassium carbonate (691mg ,5mmol) were added to degas by nitrogen flow for 15 min. At the same time, another flask was added with 5ml DMF and 1ml water and degassed by a nitrogen stream for 15 minutes. Then the flask containing the reactants was added. The reaction mixture was transferred to an oil bath and heated to 120°C for 5h. After the reaction, the solvent was removed under vacuum distillation. Chromatography of the residue on silica gel using petroleum ether/dichloromethane (v/v, 2:1)to obtain 110mg yellow solid(26% yield) ^{1}H NMR (400 MHz, $CDCl_3$) δ 10.31 (s, 2H), 9.24 (s, 1H), 7.64 (d, J = 7.9 Hz, 2H), 7.47 (d, J = 8.2 Hz,2H), 7.29 (d, J = 7.9Hz, 2H), 7.16 (t, J = 7.5Hz, 2H), 6.87 (d, J = 1.6Hz, 2H), 3.69 (t, J = 7.3Hz, 2H), 1.73 (dt, J = 15.0, 7.6Hz, 2H), 1.43 -- 1.31 (m, 6H), 0.94-0.86 (m, 3H);^{13}C NMR (101 MHz, $CDCl_3$) δ 165.47, 137.00, 128.14, 126.77, 125.90, 124.18, 121.02, 120.77, 115.34, 111.68, 99.58, 38.70,31.47, 28.88, 26.67, 22.57, 14.07.

2. NMR Spectra of all the synthesized compounds

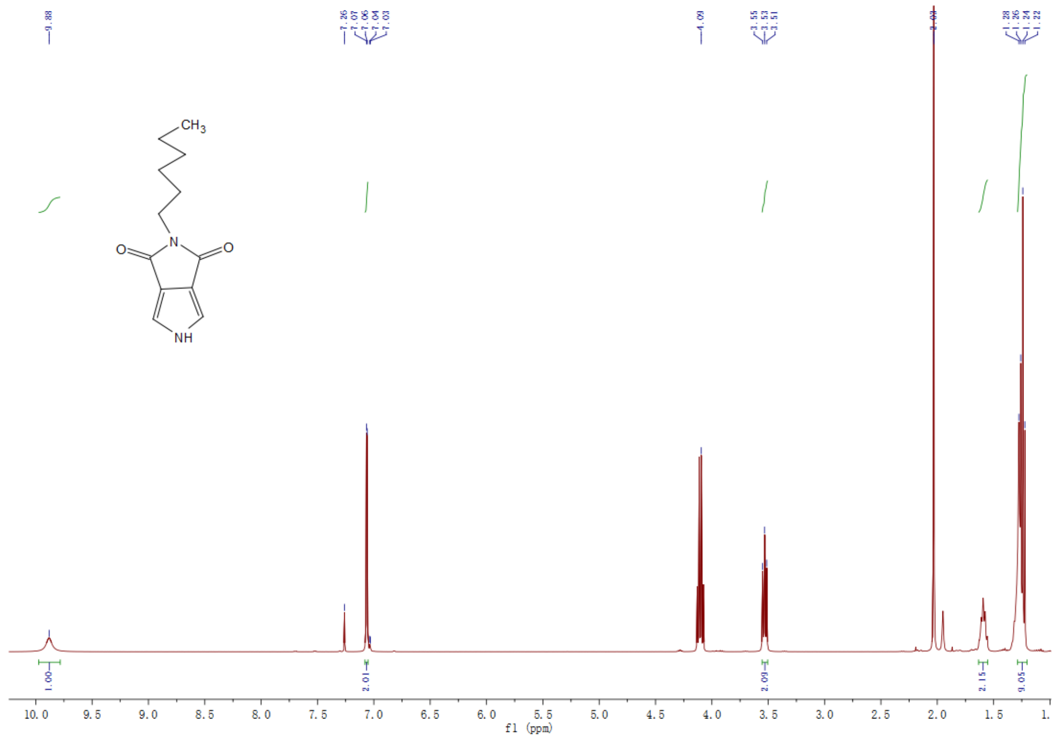

Figure S1. ^{1}H NMR spectrum of compound 2.

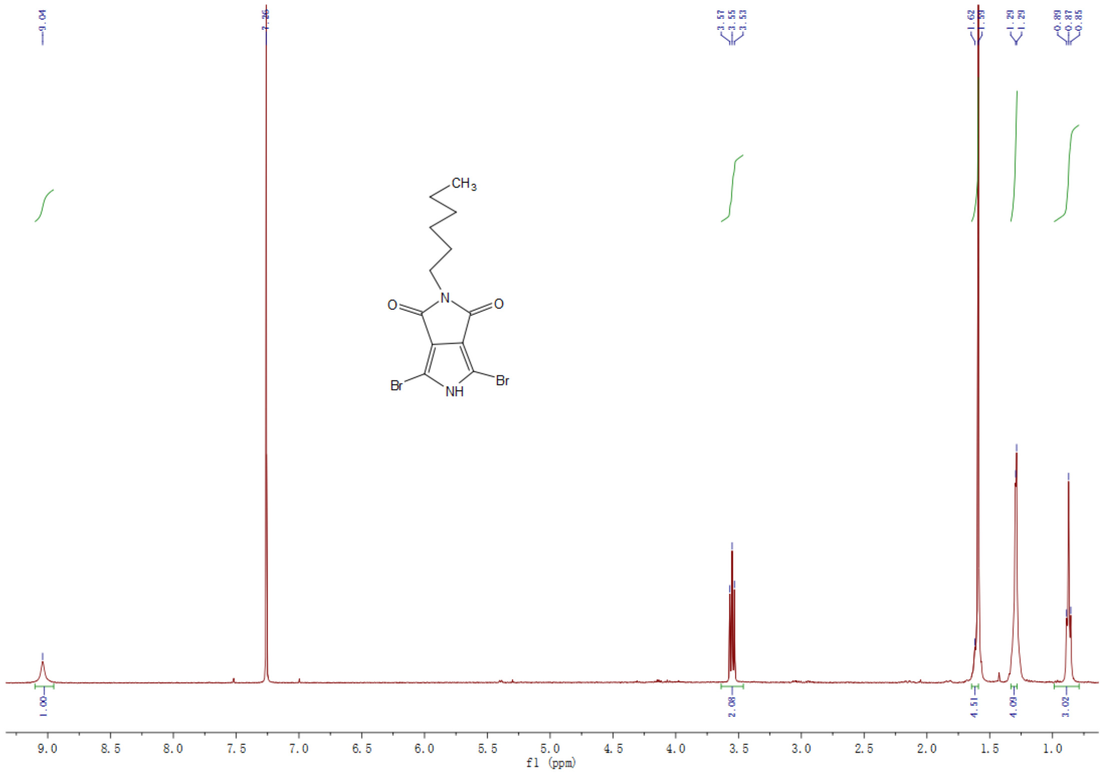

Figure S2. ^{1}H NMR spectrum of compound 3.

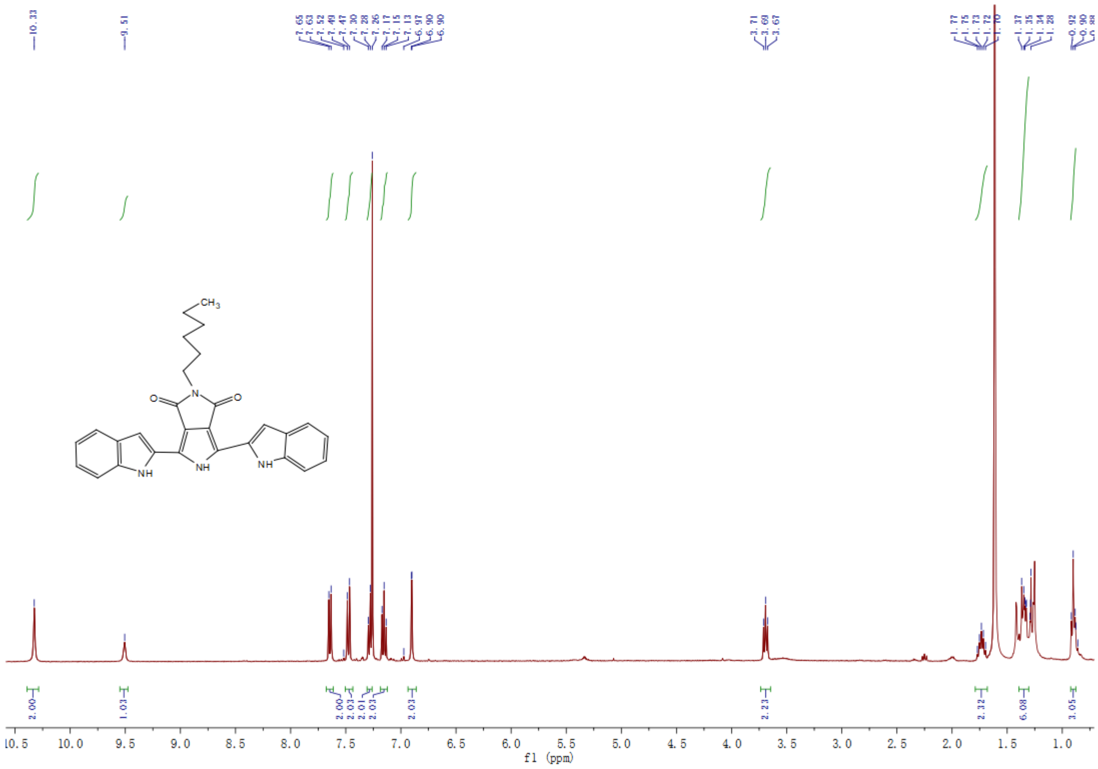

Figure S3. ^{1}H NMR spectrum of BIPPD.

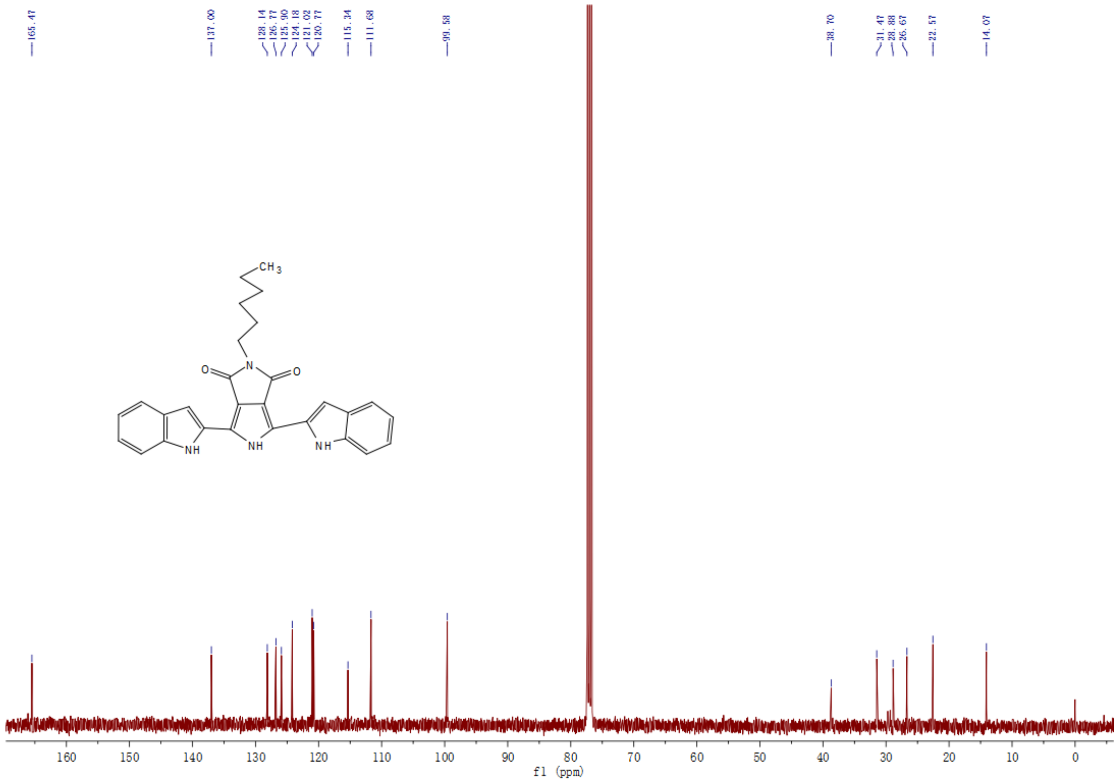

Figure S4. ^{13}C NMR spectrum of BIPPD.

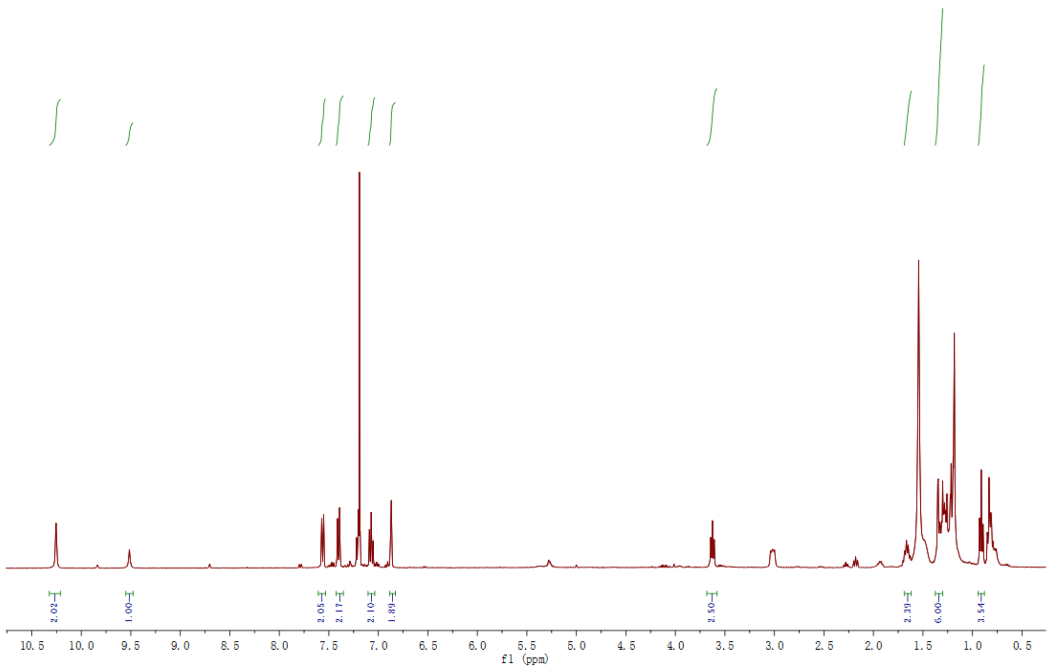

Figure S5. ^{1}H NMR spectrum of BIPPD added with 1 equivalent of PF_6^-.

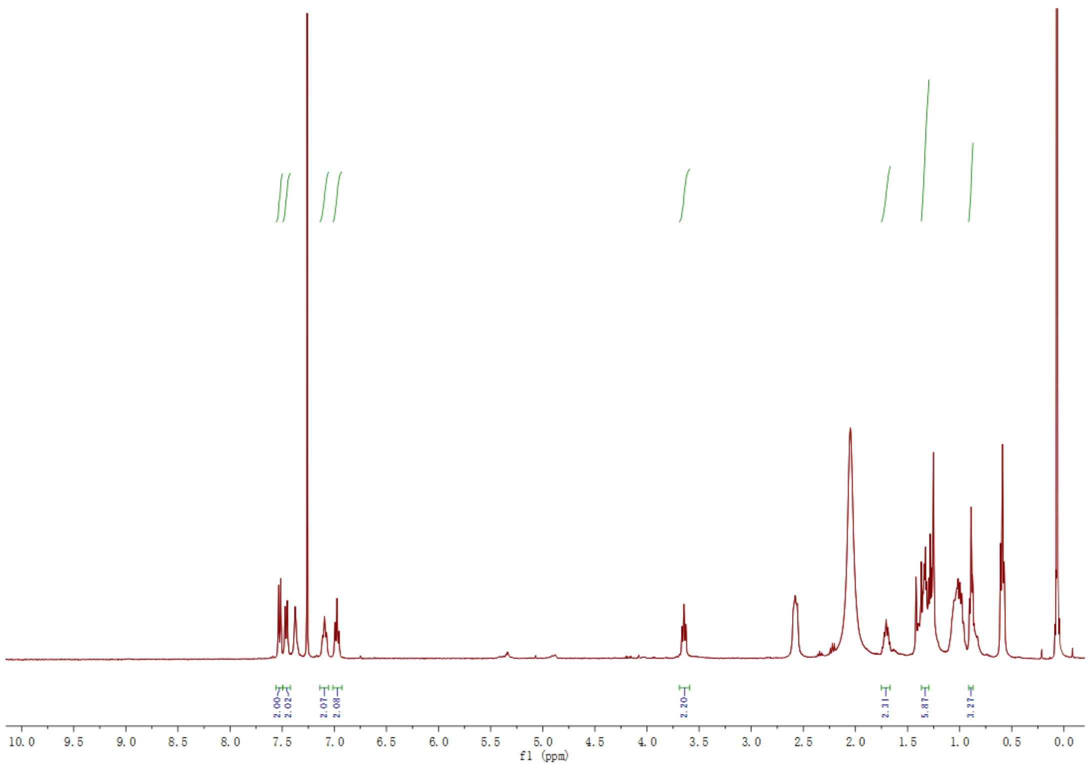

Figure S6. ^{1}H NMR spectrum of BIPPD added with 1 equivalent of TBA-H_2PO_4.

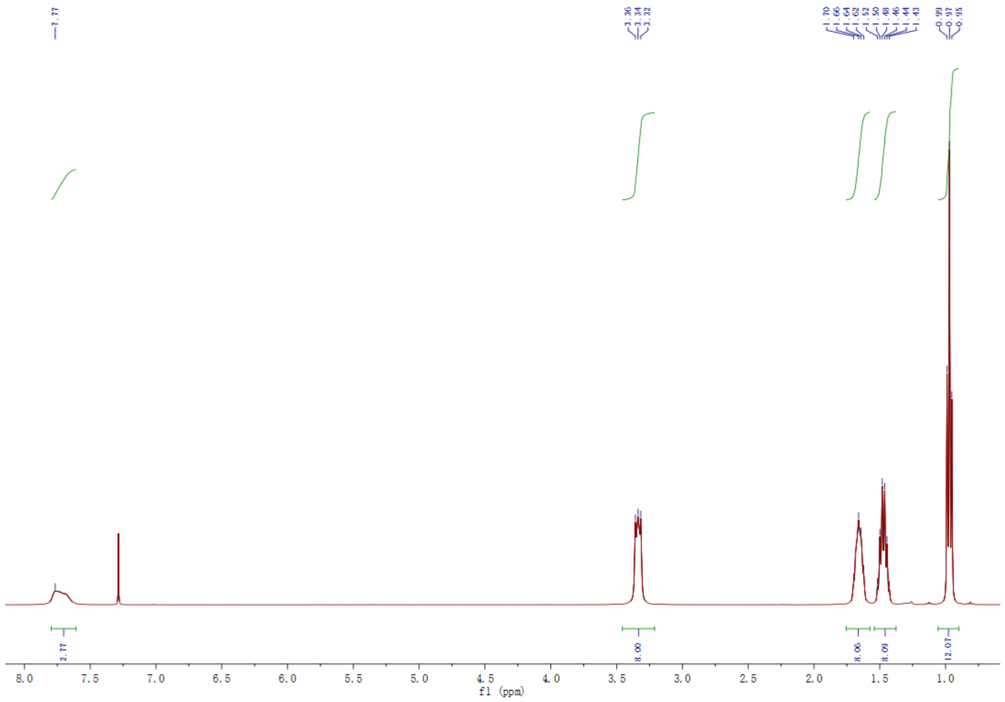

Figure S7. ^{1}H NMR spectrum of TBA-H_2PO_4.

3. ECL Efficiency Calculations

ECL quantum efficiency (Φ_x) was calculated relative to 1mM $Ru(bpy)_3(PF_6)_2$ in MeCN in the annihilation pathway whose ECL efficiency was taken as 100%. This was done by taking the sum of the integration of both the ECL intensity and current values (versus time) for the compound against the standard, as described in the following equation S1:

$$\Phi = 100\% \frac{\left[\dfrac{\int_b^a ECL\,dt}{\int_b^a Current\,dt}\right]_x}{\left[\dfrac{\int_b^a ECL\,dt}{\int_b^a Current\,dt}\right]_{Ru(bpy)_3{}^{2+}}} \qquad (S1)$$

where x represented studied sample. Equation S1 is based on the principle of generated photos per electron.

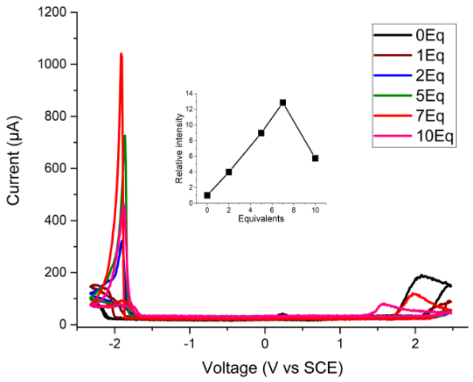

Figure S8. The corresponding ECL-voltage curves of 1mM BIPPD doped with various equivalents of $H_2PO_4^-$ in anhydrous acetonitrile with 0.1 M $TBAPF_6$ as the supporting electrolyte at a scan rate of 0.1 V/s. Inset indicates the relative enhancement result of doping different equivalents of $H_2PO_4^-$.

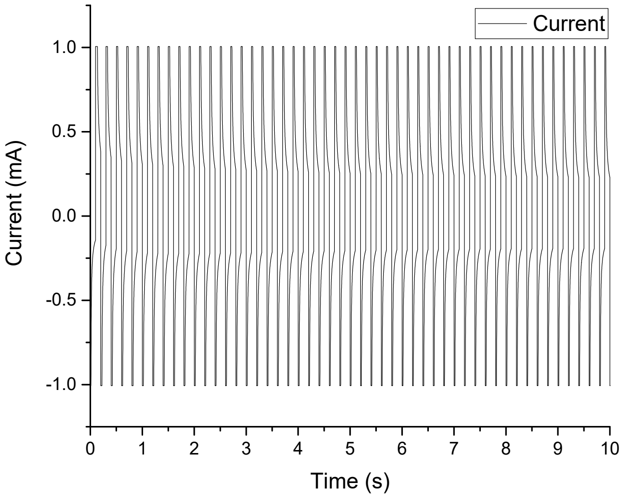

Figure S9. A small time window of a 300 s pulsing experiment using 1 mM BIPPD doped with 7 equivalents $H_2PO_4^-$ in anhydrous acetonitrile with 0.1 M $TBAPF_6$ as the supporting electrolyte with the applied potential being pulsed between -1.43 and 1.47 V vs. SCE at a frequency of 5 Hz.

4. Extra absorption and PL spectra

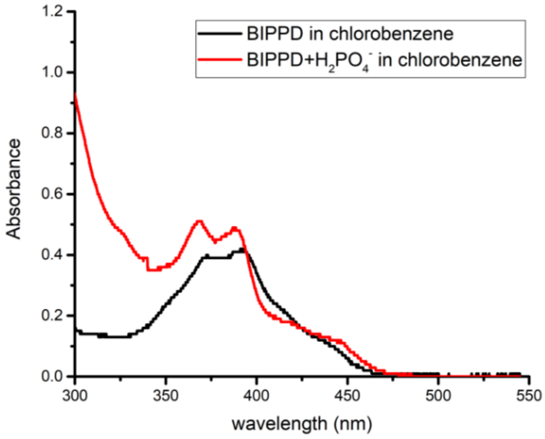

Figure S10. UV-vis absorption spectra of BIPPD (black) and BIPPD added with 3 equivalents of $H_2PO_4^-$ (red) in chlorobenzene at a concentration of 1 x10^{-5} mol/L.

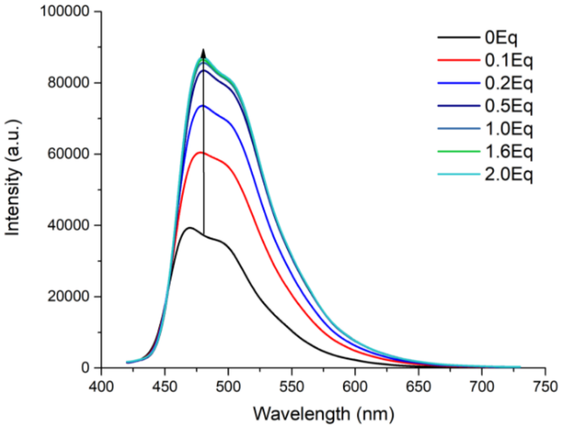

Figure S11. PL spectra of BIPPD in chlorobenzene at a concentration of 1 x10-5 mol/L (black) added with various equivalents of $H_2PO_4^-$. The excited wavelength was at 390 nm.

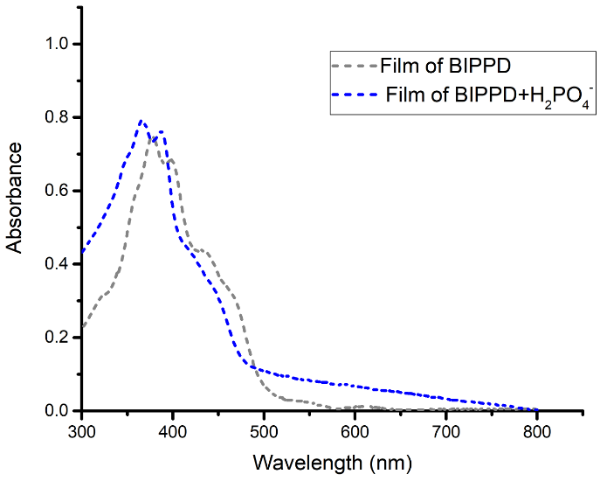

Figure S12. UV-visible spectra of BIPPD (gray, dash line) film and its film added with 3 equivalents of $H_2PO_4^-$ (blue, dash line).

Table S1. The summary of the photophysical and ECL properties of BIPPD and BIPPD/$H_2PO_4^-$ system.

System	Absorption (nm)		PL (nm)		ECL (nm)
	Solution	***Film***	*Solution*	***Film***	*Solution*
**BIPPD**	295, 372,392	196, 230, 366, 386	470,488	560	/
**BIPPD+ $H_2PO_4^-$**	290, 369,388	196, 222,375, 400	480,492	490, 509	490

5. Crystallographic Data of BIPPD

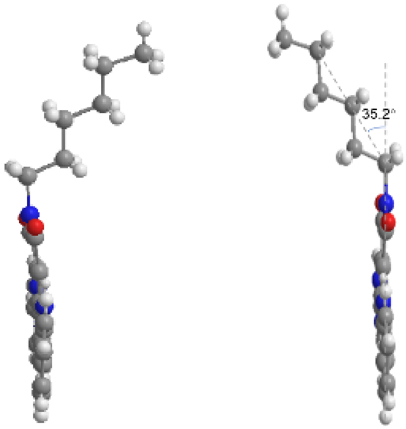

Figure S13. Side views of one conformer of BIPPD found within a unit cell of the determined crystal structure.

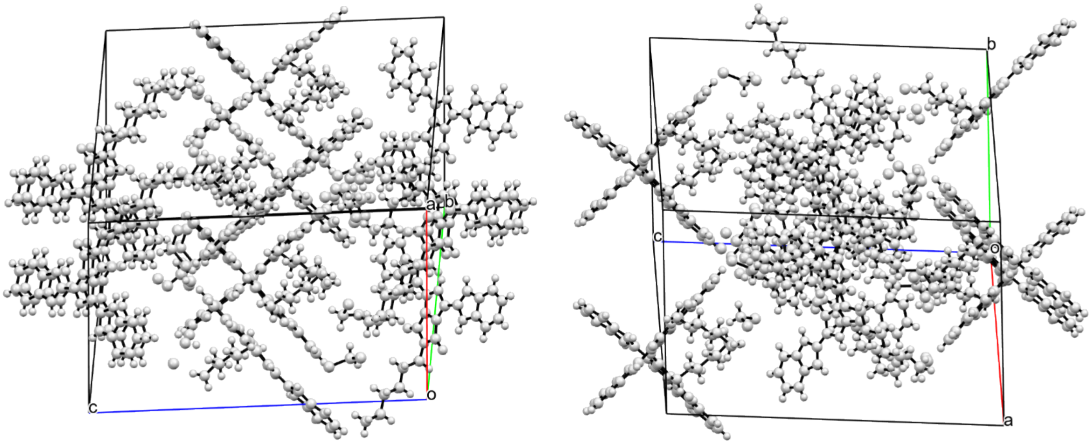

Figure S14. The X-ray crystal structure of BIPPD within the packing system in diferent views.

Table S2. Crystal data and structure refinement for BIPPD.

Identification code	BIPPD
Empirical formula	C28.75 H27.50 Cl1.50 N4 O2
Formula weight	514.22
Temperature	295(2) K
Wavelength	1.54178 A
Crystal system, space group	Monoclinic, C c
Unit cell dimensions	a = 20.4511(4) A alpha = 90 deg.
	b = 20.4148(4) A beta = 96.9050(10) deg.
	c = 25.8502(6) A gamma = 90 deg.
Volume	10714.3(4) A^3
Z, Calculated density	16, 1.275 Mg/m^3
Absorption coefficient	1.981 mm^-1
F(000)	4312
Crystal size	0.150 x 0.150 x 0.100 mm
Theta range for data collection	3.390 to 74.884 deg.

Limiting indices	-25<=h<=25, -25<=k<=25, -32<=l<=32
Reflections collected / unique	124927 / 21774 [R(int) = 0.0600]
Completeness to theta = 67.679	99.9 %
Absorption correction	Empirical (Bruker SADABS)
Max. and min. transmission	0.7538 and 0.5563
Refinement method	Full-matrix least-squares on F^2
Data / restraints / parameters	21774 / 20 / 1304
Goodness-of-fit on F^2	1.021
Final R indices [I>2sigma(I)]	R1 = 0.0585, wR2 = 0.1557
R indices (all data)	R1 = 0.0718, wR2 = 0.1731
Absolute structure parameter	0.22(3)
Extinction coefficient	0.00015(3)
Largest diff. peak and hole	0.370 and -0.482 e.A^-3

Table S3. Atomic coordinates (x 10^4) and equivalent isotropic displacement parameters (A^2 x 10^3) for BIPPD. U(eq) is defined as one third of the trace of the orthogonalized Uij tensor.

	x	y	z	U(eq)
O(1)	5320(1)	8571(2)	390(1)	49(1)
O(2)	3498(2)	9142(2)	-743(1)	57(1)
O(3)	2831(2)	3926(1)	5366(1)	46(1)
O(4)	3236(2)	5621(2)	4278(1)	56(1)
O(5)	7102(2)	4543(1)	4558(1)	48(1)
O(6)	6658(2)	2894(2)	5681(1)	55(1)
O(7)	6450(2)	7621(2)	5674(1)	56(1)
O(8)	4594(1)	7024(2)	4588(1)	47(1)
N(1)	4474(2)	8992(2)	-199(2)	45(1)
N(2)	3514(2)	7247(2)	171(1)	39(1)
N(3)	5142(2)	7224(2)	980(2)	45(1)
N(4)	2433(2)	8141(2)	-853(1)	43(1)
N(5)	3164(2)	4703(2)	4796(2)	44(1)
N(6)	1490(2)	5706(2)	5206(1)	41(1)
N(7)	1629(2)	4173(2)	5990(2)	47(1)
N(8)	2178(2)	6663(2)	4182(2)	55(1)
N(9)	6753(2)	3796(2)	5147(2)	44(1)
N(10)	8421(2)	2741(2)	4776(1)	39(1)
N(11)	8337(2)	4256(2)	3970(2)	48(1)
N(12)	7663(2)	1827(2)	5795(2)	44(1)
N(13)	5455(2)	7462(2)	5152(2)	44(1)
N(14)	6433(2)	5745(2)	4735(1)	38(1)
N(15)	7525(2)	6626(2)	5754(2)	45(1)
N(16)	4782(2)	5696(2)	3964(2)	47(1)
C(1)	4764(2)	8526(2)	148(2)	41(1)

C(2)	4276(2)	8008(2)	149(2)	40(1)
C(3)	4141(2)	7423(2)	389(2)	37(1)
C(4)	3844(2)	8810(2)	-430(2)	44(1)
C(5)	3728(2)	8176(2)	-205(2)	40(1)
C(6)	3256(2)	7699(2)	-190(2)	38(1)
C(7)	4511(2)	7047(2)	789(2)	40(1)
C(8)	4331(2)	6508(2)	1060(2)	49(1)
C(9)	4871(2)	6350(2)	1438(2)	46(1)
C(10)	4989(3)	5866(2)	1827(2)	60(1)
C(11)	5579(3)	5855(3)	2129(2)	65(1)
C(12)	6070(3)	6311(3)	2059(2)	67(1)
C(13)	5981(2)	6788(3)	1682(2)	57(1)
C(14)	5374(2)	6807(2)	1378(2)	43(1)
C(15)	2617(2)	7657(2)	-491(2)	40(1)
C(16)	2112(2)	7204(2)	-497(2)	42(1)
C(17)	1597(2)	7424(2)	-875(2)	43(1)
C(18)	957(2)	7201(2)	-1049(2)	52(1)
C(19)	567(2)	7563(3)	-1413(2)	60(1)
C(20)	793(2)	8139(3)	-1621(2)	60(1)
C(21)	1420(2)	8372(3)	-1466(2)	51(1)
C(22)	1813(2)	8009(2)	-1092(2)	42(1)
C(23)	4813(2)	9582(2)	-356(2)	52(1)
C(24)	5193(2)	9449(3)	-811(2)	58(1)
C(25)	5634(3)	10013(3)	-940(2)	68(2)
C(26)	6039(3)	9834(4)	-1378(3)	85(2)
C(27)	6527(4)	10351(5)	-1486(4)	112(3)
C(28)	6961(5)	10102(7)	-1899(5)	152(3)
C(29)	2754(2)	4452(2)	5142(2)	39(1)
C(30)	2240(2)	4940(2)	5160(2)	39(1)
C(31)	1698(2)	5104(2)	5411(2)	39(1)
C(32)	2948(2)	5310(2)	4583(2)	42(1)
C(33)	2352(2)	5451(2)	4818(2)	39(1)
C(34)	1881(2)	5926(2)	4845(2)	40(1)
C(35)	1402(2)	4776(2)	5810(2)	42(1)
C(36)	916(2)	4979(2)	6100(2)	54(1)
C(37)	855(2)	4493(2)	6482(2)	54(1)
C(38)	461(3)	4420(3)	6887(3)	79(2)
C(39)	538(4)	3883(4)	7197(3)	87(2)
C(40)	991(4)	3391(3)	7120(2)	81(2)
C(41)	1377(3)	3432(3)	6716(2)	64(1)
C(42)	1304(2)	3992(2)	6405(2)	48(1)
C(43)	1778(2)	6523(2)	4558(2)	44(1)
C(44)	1319(2)	7014(2)	4563(2)	57(1)
C(45)	1447(3)	7473(2)	4182(3)	70(2)

C(46)	1174(4)	8082(3)	4001(3)	94(2)
C(47)	1469(5)	8396(3)	3596(4)	105(3)
C(48)	1998(7)	8156(4)	3392(4)	125(4)
C(49)	2276(5)	7572(3)	3555(3)	94(2)
C(50)	1988(3)	7245(2)	3943(2)	68(2)
C(51)	3736(2)	4351(2)	4638(2)	49(1)
C(52)	3540(3)	3913(2)	4170(2)	55(1)
C(53)	4103(3)	3491(3)	4033(3)	70(2)
C(54)	3883(4)	3020(4)	3581(3)	94(2)
C(55)	4420(6)	2540(6)	3477(6)	166(6)
C(56)	4198(9)	2053(8)	3083(9)	225(9)
C(57)	7173(2)	4016(2)	4798(2)	40(1)
C(58)	7679(2)	3522(2)	4797(2)	38(1)
C(59)	8222(2)	3344(2)	4559(2)	37(1)
C(60)	6957(2)	3188(2)	5372(2)	40(1)
C(61)	7551(2)	3024(2)	5151(2)	40(1)
C(62)	8017(2)	2541(2)	5136(2)	37(1)
C(63)	8539(2)	3646(2)	4154(2)	39(1)
C(64)	9019(2)	3423(2)	3882(2)	50(1)
C(65)	9126(2)	3904(2)	3506(2)	50(1)
C(66)	9537(3)	3945(3)	3107(2)	69(1)
C(67)	9515(4)	4493(3)	2807(2)	81(2)
C(68)	9087(4)	5002(3)	2885(2)	80(2)
C(69)	8675(3)	4981(3)	3264(2)	69(2)
C(70)	8689(2)	4420(2)	3571(2)	49(1)
C(71)	8102(2)	1951(2)	5438(2)	39(1)
C(72)	8543(2)	1443(2)	5452(2)	43(1)
C(73)	8380(2)	984(2)	5834(2)	44(1)
C(74)	8622(2)	373(2)	6016(2)	52(1)
C(75)	8313(3)	52(2)	6381(2)	58(1)
C(76)	7760(3)	312(3)	6580(2)	59(1)
C(77)	7508(2)	913(2)	6411(2)	52(1)
C(78)	7827(2)	1244(2)	6040(2)	42(1)
C(79)	6170(2)	4145(2)	5272(2)	50(1)
C(80)	6319(2)	4611(2)	5728(2)	52(1)
C(81)	5736(2)	5051(2)	5809(2)	55(1)
C(82)	5900(3)	5550(3)	6235(2)	64(1)
C(83)	5340(4)	6010(3)	6324(3)	82(2)
C(84)	5504(6)	6515(4)	6730(4)	114(3)
C(85)	6098(2)	7284(2)	5368(2)	41(1)
C(86)	6214(2)	6655(2)	5127(2)	38(1)
C(87)	6697(2)	6192(2)	5103(2)	37(1)
C(88)	5161(2)	6995(2)	4805(2)	37(1)
C(89)	5659(2)	6485(2)	4782(2)	37(1)

C(90)	5797(2)	5913(2)	4533(2)	38(1)
C(91)	7337(2)	6139(2)	5398(2)	39(1)
C(92)	7825(2)	5683(2)	5413(2)	46(1)
C(93)	8345(2)	5891(2)	5793(2)	46(1)
C(94)	8972(2)	5643(3)	5983(2)	60(1)
C(95)	9356(3)	5994(3)	6358(2)	68(1)
C(96)	9141(3)	6585(3)	6559(2)	69(2)
C(97)	8531(3)	6833(3)	6382(2)	61(1)
C(98)	8140(2)	6486(2)	6004(2)	47(1)
C(99)	5430(2)	5549(2)	4122(2)	40(1)
C(100)	5629(2)	5072(2)	3804(2)	52(1)
C(101)	5083(2)	4922(2)	3427(2)	50(1)
C(102)	4981(3)	4496(3)	2995(2)	70(2)
C(103)	4374(4)	4475(3)	2706(2)	76(2)
C(104)	3862(3)	4864(4)	2840(2)	78(2)
C(105)	3940(3)	5293(3)	3255(2)	66(1)
C(106)	4559(2)	5316(2)	3545(2)	48(1)
C(107)	5126(2)	8052(2)	5307(2)	50(1)
C(108)	4757(2)	7942(2)	5773(2)	54(1)
C(109)	4324(3)	8513(3)	5886(2)	64(1)
C(110)	3908(3)	8375(4)	6318(3)	82(2)
C(111)	3416(5)	8922(5)	6409(4)	121(3)
C(112)	2947(5)	8740(8)	6778(5)	152(3)
C(113)	8792(12)	1674(9)	2409(8)	132(3)
Cl(1A)	8867(3)	2369(3)	2806(3)	153(2)
Cl(2A)	7971(3)	1483(6)	2215(6)	158(3)
C(114)	8916(17)	1840(20)	2437(11)	132(3)
Cl(1B)	8868(6)	1978(7)	3107(5)	153(2)
Cl(2B)	8201(9)	1514(15)	2173(14)	158(3)
C(115)	7200(30)	5124(19)	2272(14)	132(3)
Cl(3A)	7011(9)	4910(10)	2892(8)	184(3)
Cl(4A)	7666(11)	4668(12)	1943(9)	215(3)
C(116)	7576(8)	4113(6)	2194(6)	132(3)
Cl(3B)	7089(3)	4675(3)	2512(3)	184(3)
Cl(4B)	7270(4)	3381(4)	2109(3)	215(3)
C(117)	6516(6)	1937(5)	7642(4)	132(3)
Cl(5)	7270(2)	2134(2)	7472(2)	180(2)
Cl(6)	6045(2)	1474(2)	7201(2)	160(1)

Table S4. Bond lengths [A] and angles [deg] for BIPPD.

O(1)-C(1)	1.233(5)
O(2)-C(4)	1.215(5)
O(3)-C(29)	1.221(5)
O(4)-C(32)	1.217(5)
O(5)-C(57)	1.242(5)
O(6)-C(60)	1.219(5)
O(7)-C(85)	1.217(5)
O(8)-C(88)	1.228(5)
N(1)-C(1)	1.391(5)
N(1)-C(4)	1.405(5)
N(1)-C(23)	1.469(5)
N(2)-C(6)	1.372(5)
N(2)-C(3)	1.385(5)
N(2)-H(2A)	0.8600
N(3)-C(7)	1.373(5)
N(3)-C(14)	1.376(6)
N(3)-H(3A)	0.8600
N(4)-C(22)	1.369(5)
N(4)-C(15)	1.382(6)
N(4)-H(4A)	0.8600
N(5)-C(29)	1.393(5)
N(5)-C(32)	1.406(5)
N(5)-C(51)	1.472(5)
N(6)-C(34)	1.375(6)
N(6)-C(31)	1.385(5)
N(6)-H(6A)	0.8600
N(7)-C(35)	1.376(5)
N(7)-C(42)	1.379(6)
N(7)-H(7A)	0.8600
N(8)-C(43)	1.373(6)
N(8)-C(50)	1.374(6)
N(8)-H(8B)	0.8600
N(9)-C(57)	1.392(6)
N(9)-C(60)	1.412(5)
N(9)-C(79)	1.458(5)
N(10)-C(62)	1.377(5)
N(10)-C(59)	1.393(5)
N(10)-H(10M)	0.8600
N(11)-C(70)	1.366(6)
N(11)-C(63)	1.380(5)
N(11)-H(11I)	0.8600
N(12)-C(78)	1.370(5)

N(12)-C(71)	1.385(5)
N(12)-H(12B)	0.8600
N(13)-C(88)	1.394(5)
N(13)-C(85)	1.413(5)
N(13)-C(107)	1.459(5)
N(14)-C(87)	1.380(5)
N(14)-C(90)	1.385(5)
N(14)-H(14A)	0.8600
N(15)-C(98)	1.372(5)
N(15)-C(91)	1.378(6)
N(15)-H(15A)	0.8600
N(16)-C(106)	1.367(6)
N(16)-C(99)	1.372(5)
N(16)-H(16B)	0.8600
C(1)-C(2)	1.455(5)
C(2)-C(3)	1.388(5)
C(2)-C(5)	1.402(6)
C(3)-C(7)	1.429(6)
C(4)-C(5)	1.450(6)
C(5)-C(6)	1.374(5)
C(6)-C(15)	1.440(5)
C(7)-C(8)	1.376(6)
C(8)-C(9)	1.419(6)
C(8)-H(8A)	0.9300
C(9)-C(10)	1.410(6)
C(9)-C(14)	1.413(6)
C(10)-C(11)	1.356(8)
C(10)-H(10A)	0.9300
C(11)-C(12)	1.397(9)
C(11)-H(11A)	0.9300
C(12)-C(13)	1.374(8)
C(12)-H(12A)	0.9300
C(13)-C(14)	1.387(6)
C(13)-H(13A)	0.9300
C(15)-C(16)	1.385(6)
C(16)-C(17)	1.421(6)
C(16)-H(16A)	0.9300
C(17)-C(18)	1.407(6)
C(17)-C(22)	1.413(6)
C(18)-C(19)	1.374(7)
C(18)-H(18A)	0.9300
C(19)-C(20)	1.395(8)
C(19)-H(19A)	0.9300
C(20)-C(21)	1.380(7)

C(20)-H(20A)	0.9300
C(21)-C(22)	1.394(6)
C(21)-H(21A)	0.9300
C(23)-C(24)	1.511(8)
C(23)-H(23A)	0.9700
C(23)-H(23B)	0.9700
C(24)-C(25)	1.524(7)
C(24)-H(24A)	0.9700
C(24)-H(24B)	0.9700
C(25)-C(26)	1.526(9)
C(25)-H(25A)	0.9700
C(25)-H(25B)	0.9700
C(26)-C(27)	1.501(10)
C(26)-H(26A)	0.9700
C(26)-H(26B)	0.9700
C(27)-C(28)	1.555(14)
C(27)-H(27A)	0.9700
C(27)-H(27B)	0.9700
C(28)-H(28A)	0.9600
C(28)-H(28B)	0.9600
C(28)-H(28C)	0.9600
C(29)-C(30)	1.454(5)
C(30)-C(31)	1.391(5)
C(30)-C(33)	1.403(6)
C(31)-C(35)	1.425(6)
C(32)-C(33)	1.455(6)
C(33)-C(34)	1.376(5)
C(34)-C(43)	1.429(6)
C(35)-C(36)	1.379(6)
C(36)-C(37)	1.414(7)
C(36)-H(36A)	0.9300
C(37)-C(38)	1.404(7)
C(37)-C(42)	1.405(7)
C(38)-C(39)	1.356(10)
C(38)-H(38A)	0.9300
C(39)-C(40)	1.397(11)
C(39)-H(39A)	0.9300
C(40)-C(41)	1.384(9)
C(40)-H(40A)	0.9300
C(41)-C(42)	1.394(7)
C(41)-H(41A)	0.9300
C(43)-C(44)	1.373(6)
C(44)-C(45)	1.407(9)
C(44)-H(44A)	0.9300

C(45)-C(50)	1.409(10)
C(45)-C(46)	1.420(8)
C(46)-C(47)	1.422(13)
C(46)-H(46A)	0.9300
C(47)-C(48)	1.352(15)
C(47)-H(47A)	0.9300
C(48)-C(49)	1.367(11)
C(48)-H(48A)	0.9300
C(49)-C(50)	1.394(10)
C(49)-H(49A)	0.9300
C(51)-C(52)	1.520(7)
C(51)-H(51A)	0.9700
C(51)-H(51B)	0.9700
C(52)-C(53)	1.515(7)
C(52)-H(52A)	0.9700
C(52)-H(52B)	0.9700
C(53)-C(54)	1.538(10)
C(53)-H(53A)	0.9700
C(53)-H(53B)	0.9700
C(54)-C(55)	1.519(12)
C(54)-H(54A)	0.9700
C(54)-H(54B)	0.9700
C(55)-C(56)	1.455(18)
C(55)-H(55A)	0.9700
C(55)-H(55B)	0.9700
C(56)-H(56A)	0.9600
C(56)-H(56B)	0.9600
C(56)-H(56C)	0.9600
C(57)-C(58)	1.445(5)
C(58)-C(59)	1.383(5)
C(58)-C(61)	1.411(6)
C(59)-C(63)	1.434(6)
C(60)-C(61)	1.444(5)
C(61)-C(62)	1.375(5)
C(62)-C(71)	1.436(6)
C(63)-C(64)	1.354(6)
C(64)-C(65)	1.417(7)
C(64)-H(64A)	0.9300
C(65)-C(70)	1.403(7)
C(65)-C(66)	1.408(7)
C(66)-C(67)	1.360(9)
C(66)-H(66A)	0.9300
C(67)-C(68)	1.389(10)
C(67)-H(67A)	0.9300

C(68)-C(69)	1.368(9)
C(68)-H(68A)	0.9300
C(69)-C(70)	1.392(7)
C(69)-H(69A)	0.9300
C(71)-C(72)	1.371(6)
C(72)-C(73)	1.431(6)
C(72)-H(72A)	0.9300
C(73)-C(74)	1.403(6)
C(73)-C(78)	1.409(6)
C(74)-C(75)	1.365(7)
C(74)-H(74A)	0.9300
C(75)-C(76)	1.401(8)
C(75)-H(75A)	0.9300
C(76)-C(77)	1.382(7)
C(76)-H(76A)	0.9300
C(77)-C(78)	1.397(6)
C(77)-H(77A)	0.9300
C(79)-C(80)	1.519(7)
C(79)-H(79A)	0.9700
C(79)-H(79B)	0.9700
C(80)-C(81)	1.527(6)
C(80)-H(80A)	0.9700
C(80)-H(80B)	0.9700
C(81)-C(82)	1.508(8)
C(81)-H(81A)	0.9700
C(81)-H(81B)	0.9700
C(82)-C(83)	1.519(8)
C(82)-H(82A)	0.9700
C(82)-H(82B)	0.9700
C(83)-C(84)	1.481(11)
C(83)-H(83A)	0.9700
C(83)-H(83B)	0.9700
C(84)-H(84A)	0.9600
C(84)-H(84B)	0.9600
C(84)-H(84C)	0.9600
C(85)-C(86)	1.459(5)
C(86)-C(87)	1.374(5)
C(86)-C(89)	1.400(6)
C(87)-C(91)	1.439(5)
C(88)-C(89)	1.463(5)
C(89)-C(90)	1.378(5)
C(90)-C(99)	1.434(6)
C(91)-C(92)	1.362(6)
C(92)-C(93)	1.424(7)

C(92)-H(92A)	0.9300
C(93)-C(94)	1.409(6)
C(93)-C(98)	1.416(7)
C(94)-C(95)	1.372(8)
C(94)-H(94A)	0.9300
C(95)-C(96)	1.406(9)
C(95)-H(95A)	0.9300
C(96)-C(97)	1.374(8)
C(96)-H(96A)	0.9300
C(97)-C(98)	1.381(7)
C(97)-H(97A)	0.9300
C(99)-C(100)	1.368(6)
C(100)-C(101)	1.423(7)
C(100)-H(10B)	0.9300
C(101)-C(106)	1.402(7)
C(101)-C(102)	1.412(7)
C(102)-C(103)	1.371(9)
C(102)-H(10C)	0.9300
C(103)-C(104)	1.391(10)
C(103)-H(10D)	0.9300
C(104)-C(105)	1.378(9)
C(104)-H(10E)	0.9300
C(105)-C(106)	1.392(7)
C(105)-H(10F)	0.9300
C(107)-C(108)	1.514(7)
C(107)-H(10G)	0.9700
C(107)-H(10H)	0.9700
C(108)-C(109)	1.513(7)
C(108)-H(10I)	0.9700
C(108)-H(10J)	0.9700
C(109)-C(110)	1.511(9)
C(109)-H(10K)	0.9700
C(109)-H(10L)	0.9700
C(110)-C(111)	1.540(11)
C(110)-H(11B)	0.9700
C(110)-H(11C)	0.9700
C(111)-C(112)	1.479(13)
C(111)-H(11D)	0.9700
C(111)-H(11E)	0.9700
C(112)-H(11F)	0.9600
C(112)-H(11G)	0.9600
C(112)-H(11H)	0.9600
C(113)-Cl(2A)	1.74(2)
C(113)-Cl(1A)	1.747(19)

C(113)-H(11I)	0.9700
C(113)-H(11J)	0.9700
C(114)-Cl(2B)	1.68(2)
C(114)-Cl(1B)	1.77(2)
C(114)-H(11K)	0.9700
C(114)-H(11L)	0.9700
C(115)-Cl(4A)	1.64(2)
C(115)-Cl(3A)	1.75(2)
C(115)-H(11M)	0.9700
C(115)-H(11N)	0.9700
C(116)-Cl(4B)	1.625(14)
C(116)-Cl(3B)	1.785(13)
C(116)-H(11O)	0.9700
C(116)-H(11P)	0.9700
C(117)-Cl(6)	1.691(12)
C(117)-Cl(5)	1.700(11)
C(117)-H(11Q)	0.9700
C(117)-H(11R)	0.9700

C(1)-N(1)-C(4)	113.0(3)
C(1)-N(1)-C(23)	124.0(4)
C(4)-N(1)-C(23)	122.6(4)
C(6)-N(2)-C(3)	111.3(3)
C(6)-N(2)-H(2A)	124.3
C(3)-N(2)-H(2A)	124.3
C(7)-N(3)-C(14)	109.3(3)
C(7)-N(3)-H(3A)	125.3
C(14)-N(3)-H(3A)	125.3
C(22)-N(4)-C(15)	109.2(3)
C(22)-N(4)-H(4A)	125.4
C(15)-N(4)-H(4A)	125.4
C(29)-N(5)-C(32)	113.1(3)
C(29)-N(5)-C(51)	123.9(3)
C(32)-N(5)-C(51)	122.9(4)
C(34)-N(6)-C(31)	111.9(3)
C(34)-N(6)-H(6A)	124.0
C(31)-N(6)-H(6A)	124.0
C(35)-N(7)-C(42)	109.2(3)
C(35)-N(7)-H(7A)	125.4
C(42)-N(7)-H(7A)	125.4
C(43)-N(8)-C(50)	109.7(4)
C(43)-N(8)-H(8B)	125.1
C(50)-N(8)-H(8B)	125.1
C(57)-N(9)-C(60)	112.1(3)

C(57)-N(9)-C(79)	124.8(3)
C(60)-N(9)-C(79)	123.1(4)
C(62)-N(10)-C(59)	111.4(3)
C(62)-N(10)-H(10M)	124.3
C(59)-N(10)-H(10M)	124.3
C(70)-N(11)-C(63)	108.6(4)
C(70)-N(11)-H(11I)	125.7
C(63)-N(11)-H(11I)	125.7
C(78)-N(12)-C(71)	108.9(3)
C(78)-N(12)-H(12B)	125.6
C(71)-N(12)-H(12B)	125.6
C(88)-N(13)-C(85)	112.7(3)
C(88)-N(13)-C(107)	124.4(3)
C(85)-N(13)-C(107)	122.8(4)
C(87)-N(14)-C(90)	111.6(3)
C(87)-N(14)-H(14A)	124.2
C(90)-N(14)-H(14A)	124.2
C(98)-N(15)-C(91)	109.3(3)
C(98)-N(15)-H(15A)	125.4
C(91)-N(15)-H(15A)	125.4
C(106)-N(16)-C(99)	109.2(4)
C(106)-N(16)-H(16B)	125.4
C(99)-N(16)-H(16B)	125.4
O(1)-C(1)-N(1)	124.8(4)
O(1)-C(1)-C(2)	130.2(4)
N(1)-C(1)-C(2)	105.0(3)
C(3)-C(2)-C(5)	108.5(3)
C(3)-C(2)-C(1)	143.1(4)
C(5)-C(2)-C(1)	108.4(3)
N(2)-C(3)-C(2)	105.4(3)
N(2)-C(3)-C(7)	122.5(3)
C(2)-C(3)-C(7)	132.1(3)
O(2)-C(4)-N(1)	124.5(4)
O(2)-C(4)-C(5)	130.9(4)
N(1)-C(4)-C(5)	104.6(3)
C(6)-C(5)-C(2)	108.5(4)
C(6)-C(5)-C(4)	142.4(4)
C(2)-C(5)-C(4)	108.9(3)
N(2)-C(6)-C(5)	106.4(3)
N(2)-C(6)-C(15)	125.2(3)
C(5)-C(6)-C(15)	128.4(4)
N(3)-C(7)-C(8)	109.0(4)
N(3)-C(7)-C(3)	120.6(4)
C(8)-C(7)-C(3)	130.4(4)

C(7)-C(8)-C(9)	107.4(4)
C(7)-C(8)-H(8A)	126.3
C(9)-C(8)-H(8A)	126.3
C(10)-C(9)-C(14)	118.4(4)
C(10)-C(9)-C(8)	134.7(4)
C(14)-C(9)-C(8)	106.9(4)
C(11)-C(10)-C(9)	119.2(5)
C(11)-C(10)-H(10A)	120.4
C(9)-C(10)-H(10A)	120.4
C(10)-C(11)-C(12)	121.2(5)
C(10)-C(11)-H(11A)	119.4
C(12)-C(11)-H(11A)	119.4
C(13)-C(12)-C(11)	121.9(5)
C(13)-C(12)-H(12A)	119.0
C(11)-C(12)-H(12A)	119.0
C(12)-C(13)-C(14)	117.1(5)
C(12)-C(13)-H(13A)	121.5
C(14)-C(13)-H(13A)	121.5
N(3)-C(14)-C(13)	130.3(4)
N(3)-C(14)-C(9)	107.4(4)
C(13)-C(14)-C(9)	122.2(4)
N(4)-C(15)-C(16)	109.1(4)
N(4)-C(15)-C(6)	118.6(3)
C(16)-C(15)-C(6)	132.3(4)
C(15)-C(16)-C(17)	106.7(4)
C(15)-C(16)-H(16A)	126.7
C(17)-C(16)-H(16A)	126.7
C(18)-C(17)-C(22)	118.1(4)
C(18)-C(17)-C(16)	134.5(4)
C(22)-C(17)-C(16)	107.4(3)
C(19)-C(18)-C(17)	119.0(5)
C(19)-C(18)-H(18A)	120.5
C(17)-C(18)-H(18A)	120.5
C(18)-C(19)-C(20)	121.7(4)
C(18)-C(19)-H(19A)	119.1
C(20)-C(19)-H(19A)	119.1
C(21)-C(20)-C(19)	121.2(5)
C(21)-C(20)-H(20A)	119.4
C(19)-C(20)-H(20A)	119.4
C(20)-C(21)-C(22)	117.2(5)
C(20)-C(21)-H(21A)	121.4
C(22)-C(21)-H(21A)	121.4
N(4)-C(22)-C(21)	129.4(4)
N(4)-C(22)-C(17)	107.7(4)

C(21)-C(22)-C(17)	122.8(4)
N(1)-C(23)-C(24)	111.8(4)
N(1)-C(23)-H(23A)	109.3
C(24)-C(23)-H(23A)	109.3
N(1)-C(23)-H(23B)	109.3
C(24)-C(23)-H(23B)	109.3
H(23A)-C(23)-H(23B)	107.9
C(23)-C(24)-C(25)	114.1(5)
C(23)-C(24)-H(24A)	108.7
C(25)-C(24)-H(24A)	108.7
C(23)-C(24)-H(24B)	108.7
C(25)-C(24)-H(24B)	108.7
H(24A)-C(24)-H(24B)	107.6
C(24)-C(25)-C(26)	111.7(6)
C(24)-C(25)-H(25A)	109.3
C(26)-C(25)-H(25A)	109.3
C(24)-C(25)-H(25B)	109.3
C(26)-C(25)-H(25B)	109.3
H(25A)-C(25)-H(25B)	107.9
C(27)-C(26)-C(25)	113.7(7)
C(27)-C(26)-H(26A)	108.8
C(25)-C(26)-H(26A)	108.8
C(27)-C(26)-H(26B)	108.8
C(25)-C(26)-H(26B)	108.8
H(26A)-C(26)-H(26B)	107.7
C(26)-C(27)-C(28)	110.1(10)
C(26)-C(27)-H(27A)	109.6
C(28)-C(27)-H(27A)	109.6
C(26)-C(27)-H(27B)	109.6
C(28)-C(27)-H(27B)	109.6
H(27A)-C(27)-H(27B)	108.2
C(27)-C(28)-H(28A)	109.5
C(27)-C(28)-H(28B)	109.5
H(28A)-C(28)-H(28B)	109.5
C(27)-C(28)-H(28C)	109.5
H(28A)-C(28)-H(28C)	109.5
H(28B)-C(28)-H(28C)	109.5
O(3)-C(29)-N(5)	125.1(3)
O(3)-C(29)-C(30)	129.8(4)
N(5)-C(29)-C(30)	105.1(3)
C(31)-C(30)-C(33)	108.3(3)
C(31)-C(30)-C(29)	143.2(4)
C(33)-C(30)-C(29)	108.4(3)
N(6)-C(31)-C(30)	105.1(3)

N(6)-C(31)-C(35)	123.8(3)
C(30)-C(31)-C(35)	131.1(4)
O(4)-C(32)-N(5)	124.1(4)
O(4)-C(32)-C(33)	131.5(4)
N(5)-C(32)-C(33)	104.4(3)
C(34)-C(33)-C(30)	108.9(4)
C(34)-C(33)-C(32)	142.0(4)
C(30)-C(33)-C(32)	109.0(3)
N(6)-C(34)-C(33)	105.7(3)
N(6)-C(34)-C(43)	124.8(4)
C(33)-C(34)-C(43)	129.5(4)
N(7)-C(35)-C(36)	108.7(4)
N(7)-C(35)-C(31)	120.6(3)
C(36)-C(35)-C(31)	130.6(4)
C(35)-C(36)-C(37)	107.3(4)
C(35)-C(36)-H(36A)	126.3
C(37)-C(36)-H(36A)	126.3
C(38)-C(37)-C(42)	118.1(5)
C(38)-C(37)-C(36)	134.4(5)
C(42)-C(37)-C(36)	107.4(4)
C(39)-C(38)-C(37)	119.4(6)
C(39)-C(38)-H(38A)	120.3
C(37)-C(38)-H(38A)	120.3
C(38)-C(39)-C(40)	121.9(6)
C(38)-C(39)-H(39A)	119.1
C(40)-C(39)-H(39A)	119.1
C(41)-C(40)-C(39)	120.8(6)
C(41)-C(40)-H(40A)	119.6
C(39)-C(40)-H(40A)	119.6
C(40)-C(41)-C(42)	117.0(6)
C(40)-C(41)-H(41A)	121.5
C(42)-C(41)-H(41A)	121.5
N(7)-C(42)-C(41)	129.9(5)
N(7)-C(42)-C(37)	107.3(4)
C(41)-C(42)-C(37)	122.8(5)
N(8)-C(43)-C(44)	108.8(4)
N(8)-C(43)-C(34)	118.8(4)
C(44)-C(43)-C(34)	132.4(5)
C(43)-C(44)-C(45)	107.1(5)
C(43)-C(44)-H(44A)	126.4
C(45)-C(44)-H(44A)	126.4
C(44)-C(45)-C(50)	108.0(4)
C(44)-C(45)-C(46)	135.7(7)
C(50)-C(45)-C(46)	116.3(7)

C(45)-C(46)-C(47)	117.0(8)
C(45)-C(46)-H(46A)	121.5
C(47)-C(46)-H(46A)	121.5
C(48)-C(47)-C(46)	123.7(7)
C(48)-C(47)-H(47A)	118.2
C(46)-C(47)-H(47A)	118.2
C(47)-C(48)-C(49)	121.2(9)
C(47)-C(48)-H(48A)	119.4
C(49)-C(48)-H(48A)	119.4
C(48)-C(49)-C(50)	116.4(9)
C(48)-C(49)-H(49A)	121.8
C(50)-C(49)-H(49A)	121.8
N(8)-C(50)-C(49)	128.1(6)
N(8)-C(50)-C(45)	106.4(5)
C(49)-C(50)-C(45)	125.5(6)
N(5)-C(51)-C(52)	111.5(4)
N(5)-C(51)-H(51A)	109.3
C(52)-C(51)-H(51A)	109.3
N(5)-C(51)-H(51B)	109.3
C(52)-C(51)-H(51B)	109.3
H(51A)-C(51)-H(51B)	108.0
C(53)-C(52)-C(51)	112.7(5)
C(53)-C(52)-H(52A)	109.1
C(51)-C(52)-H(52A)	109.1
C(53)-C(52)-H(52B)	109.1
C(51)-C(52)-H(52B)	109.1
H(52A)-C(52)-H(52B)	107.8
C(52)-C(53)-C(54)	111.9(6)
C(52)-C(53)-H(53A)	109.2
C(54)-C(53)-H(53A)	109.2
C(52)-C(53)-H(53B)	109.2
C(54)-C(53)-H(53B)	109.2
H(53A)-C(53)-H(53B)	107.9
C(55)-C(54)-C(53)	112.7(8)
C(55)-C(54)-H(54A)	109.1
C(53)-C(54)-H(54A)	109.1
C(55)-C(54)-H(54B)	109.1
C(53)-C(54)-H(54B)	109.1
H(54A)-C(54)-H(54B)	107.8
C(56)-C(55)-C(54)	113.2(12)
C(56)-C(55)-H(55A)	108.9
C(54)-C(55)-H(55A)	108.9
C(56)-C(55)-H(55B)	108.9
C(54)-C(55)-H(55B)	108.9

H(55A)-C(55)-H(55B)	107.8
C(55)-C(56)-H(56A)	109.5
C(55)-C(56)-H(56B)	109.5
H(56A)-C(56)-H(56B)	109.5
C(55)-C(56)-H(56C)	109.5
H(56A)-C(56)-H(56C)	109.5
H(56B)-C(56)-H(56C)	109.5
O(5)-C(57)-N(9)	124.0(4)
O(5)-C(57)-C(58)	130.2(4)
N(9)-C(57)-C(58)	105.8(3)
C(59)-C(58)-C(61)	108.7(3)
C(59)-C(58)-C(57)	142.9(4)
C(61)-C(58)-C(57)	108.3(4)
C(58)-C(59)-N(10)	105.3(3)
C(58)-C(59)-C(63)	132.0(4)
N(10)-C(59)-C(63)	122.6(3)
O(6)-C(60)-N(9)	123.9(4)
O(6)-C(60)-C(61)	131.0(4)
N(9)-C(60)-C(61)	105.1(3)
C(62)-C(61)-C(58)	108.4(4)
C(62)-C(61)-C(60)	142.8(4)
C(58)-C(61)-C(60)	108.7(3)
C(61)-C(62)-N(10)	106.3(3)
C(61)-C(62)-C(71)	128.7(4)
N(10)-C(62)-C(71)	125.0(3)
C(64)-C(63)-N(11)	109.2(4)
C(64)-C(63)-C(59)	130.9(4)
N(11)-C(63)-C(59)	119.9(3)
C(63)-C(64)-C(65)	107.8(4)
C(63)-C(64)-H(64A)	126.1
C(65)-C(64)-H(64A)	126.1
C(70)-C(65)-C(66)	119.2(5)
C(70)-C(65)-C(64)	106.5(4)
C(66)-C(65)-C(64)	134.3(5)
C(67)-C(66)-C(65)	119.0(6)
C(67)-C(66)-H(66A)	120.5
C(65)-C(66)-H(66A)	120.5
C(66)-C(67)-C(68)	120.7(6)
C(66)-C(67)-H(67A)	119.6
C(68)-C(67)-H(67A)	119.6
C(69)-C(68)-C(67)	122.2(5)
C(69)-C(68)-H(68A)	118.9
C(67)-C(68)-H(68A)	118.9
C(68)-C(69)-C(70)	117.5(6)

C(68)-C(69)-H(69A)	121.2
C(70)-C(69)-H(69A)	121.2
N(11)-C(70)-C(69)	130.8(5)
N(11)-C(70)-C(65)	108.0(4)
C(69)-C(70)-C(65)	121.2(5)
C(72)-C(71)-N(12)	108.8(4)
C(72)-C(71)-C(62)	133.1(4)
N(12)-C(71)-C(62)	118.1(3)
C(71)-C(72)-C(73)	107.8(4)
C(71)-C(72)-H(72A)	126.1
C(73)-C(72)-H(72A)	126.1
C(74)-C(73)-C(78)	118.4(4)
C(74)-C(73)-C(72)	135.4(4)
C(78)-C(73)-C(72)	106.1(4)
C(75)-C(74)-C(73)	118.9(4)
C(75)-C(74)-H(74A)	120.5
C(73)-C(74)-H(74A)	120.5
C(74)-C(75)-C(76)	122.3(4)
C(74)-C(75)-H(75A)	118.9
C(76)-C(75)-H(75A)	118.9
C(77)-C(76)-C(75)	120.5(5)
C(77)-C(76)-H(76A)	119.8
C(75)-C(76)-H(76A)	119.8
C(76)-C(77)-C(78)	117.3(4)
C(76)-C(77)-H(77A)	121.3
C(78)-C(77)-H(77A)	121.3
N(12)-C(78)-C(77)	128.9(4)
N(12)-C(78)-C(73)	108.4(4)
C(77)-C(78)-C(73)	122.7(4)
N(9)-C(79)-C(80)	112.7(4)
N(9)-C(79)-H(79A)	109.1
C(80)-C(79)-H(79A)	109.1
N(9)-C(79)-H(79B)	109.1
C(80)-C(79)-H(79B)	109.1
H(79A)-C(79)-H(79B)	107.8
C(79)-C(80)-C(81)	112.9(4)
C(79)-C(80)-H(80A)	109.0
C(81)-C(80)-H(80A)	109.0
C(79)-C(80)-H(80B)	109.0
C(81)-C(80)-H(80B)	109.0
H(80A)-C(80)-H(80B)	107.8
C(82)-C(81)-C(80)	112.8(5)
C(82)-C(81)-H(81A)	109.0
C(80)-C(81)-H(81A)	109.0

C(82)-C(81)-H(81B)	109.0
C(80)-C(81)-H(81B)	109.0
H(81A)-C(81)-H(81B)	107.8
C(81)-C(82)-C(83)	115.0(5)
C(81)-C(82)-H(82A)	108.5
C(83)-C(82)-H(82A)	108.5
C(81)-C(82)-H(82B)	108.5
C(83)-C(82)-H(82B)	108.5
H(82A)-C(82)-H(82B)	107.5
C(84)-C(83)-C(82)	115.3(7)
C(84)-C(83)-H(83A)	108.5
C(82)-C(83)-H(83A)	108.5
C(84)-C(83)-H(83B)	108.5
C(82)-C(83)-H(83B)	108.5
H(83A)-C(83)-H(83B)	107.5
C(83)-C(84)-H(84A)	109.5
C(83)-C(84)-H(84B)	109.5
H(84A)-C(84)-H(84B)	109.5
C(83)-C(84)-H(84C)	109.5
H(84A)-C(84)-H(84C)	109.5
H(84B)-C(84)-H(84C)	109.5
O(7)-C(85)-N(13)	124.2(4)
O(7)-C(85)-C(86)	131.3(4)
N(13)-C(85)-C(86)	104.4(3)
C(87)-C(86)-C(89)	109.4(3)
C(87)-C(86)-C(85)	141.2(4)
C(89)-C(86)-C(85)	109.3(3)
C(86)-C(87)-N(14)	105.2(3)
C(86)-C(87)-C(91)	129.9(4)
N(14)-C(87)-C(91)	124.8(3)
O(8)-C(88)-N(13)	124.8(3)
O(8)-C(88)-C(89)	129.8(4)
N(13)-C(88)-C(89)	105.4(3)
C(90)-C(89)-C(86)	108.2(3)
C(90)-C(89)-C(88)	143.5(4)
C(86)-C(89)-C(88)	108.2(3)
C(89)-C(90)-N(14)	105.6(3)
C(89)-C(90)-C(99)	131.9(3)
N(14)-C(90)-C(99)	122.4(3)
C(92)-C(91)-N(15)	109.2(4)
C(92)-C(91)-C(87)	133.2(4)
N(15)-C(91)-C(87)	117.6(3)
C(91)-C(92)-C(93)	107.5(4)
C(91)-C(92)-H(92A)	126.3

C(93)-C(92)-H(92A)	126.3
C(94)-C(93)-C(98)	118.2(5)
C(94)-C(93)-C(92)	134.9(5)
C(98)-C(93)-C(92)	106.8(4)
C(95)-C(94)-C(93)	118.6(5)
C(95)-C(94)-H(94A)	120.7
C(93)-C(94)-H(94A)	120.7
C(94)-C(95)-C(96)	122.1(5)
C(94)-C(95)-H(95A)	118.9
C(96)-C(95)-H(95A)	118.9
C(97)-C(96)-C(95)	120.2(5)
C(97)-C(96)-H(96A)	119.9
C(95)-C(96)-H(96A)	119.9
C(96)-C(97)-C(98)	118.3(5)
C(96)-C(97)-H(97A)	120.8
C(98)-C(97)-H(97A)	120.8
N(15)-C(98)-C(97)	130.2(5)
N(15)-C(98)-C(93)	107.2(4)
C(97)-C(98)-C(93)	122.6(4)
C(100)-C(99)-N(16)	109.0(4)
C(100)-C(99)-C(90)	130.3(4)
N(16)-C(99)-C(90)	120.5(4)
C(99)-C(100)-C(101)	107.3(4)
C(99)-C(100)-H(10B)	126.3
C(101)-C(100)-H(10B)	126.3
C(106)-C(101)-C(102)	118.7(5)
C(106)-C(101)-C(100)	106.6(4)
C(102)-C(101)-C(100)	134.7(5)
C(103)-C(102)-C(101)	119.3(6)
C(103)-C(102)-H(10C)	120.4
C(101)-C(102)-H(10C)	120.4
C(102)-C(103)-C(104)	120.3(5)
C(102)-C(103)-H(10D)	119.8
C(104)-C(103)-H(10D)	119.8
C(105)-C(104)-C(103)	122.5(5)
C(105)-C(104)-H(10E)	118.8
C(103)-C(104)-H(10E)	118.8
C(104)-C(105)-C(106)	117.0(5)
C(104)-C(105)-H(10F)	121.5
C(106)-C(105)-H(10F)	121.5
N(16)-C(106)-C(105)	129.9(5)
N(16)-C(106)-C(101)	107.9(4)
C(105)-C(106)-C(101)	122.2(5)
N(13)-C(107)-C(108)	112.7(4)

N(13)-C(107)-H(10G)	109.1
C(108)-C(107)-H(10G)	109.1
N(13)-C(107)-H(10H)	109.1
C(108)-C(107)-H(10H)	109.1
H(10G)-C(107)-H(10H)	107.8
C(109)-C(108)-C(107)	113.4(5)
C(109)-C(108)-H(10I)	108.9
C(107)-C(108)-H(10I)	108.9
C(109)-C(108)-H(10J)	108.9
C(107)-C(108)-H(10J)	108.9
H(10I)-C(108)-H(10J)	107.7
C(110)-C(109)-C(108)	113.1(5)
C(110)-C(109)-H(10K)	109.0
C(108)-C(109)-H(10K)	109.0
C(110)-C(109)-H(10L)	109.0
C(108)-C(109)-H(10L)	109.0
H(10K)-C(109)-H(10L)	107.8
C(109)-C(110)-C(111)	114.4(7)
C(109)-C(110)-H(11B)	108.7
C(111)-C(110)-H(11B)	108.7
C(109)-C(110)-H(11C)	108.7
C(111)-C(110)-H(11C)	108.7
H(11B)-C(110)-H(11C)	107.6
C(112)-C(111)-C(110)	113.7(10)
C(112)-C(111)-H(11D)	108.8
C(110)-C(111)-H(11D)	108.8
C(112)-C(111)-H(11E)	108.8
C(110)-C(111)-H(11E)	108.8
H(11D)-C(111)-H(11E)	107.7
C(111)-C(112)-H(11F)	109.5
C(111)-C(112)-H(11G)	109.5
H(11F)-C(112)-H(11G)	109.5
C(111)-C(112)-H(11H)	109.5
H(11F)-C(112)-H(11H)	109.5
H(11G)-C(112)-H(11H)	109.5
Cl(2A)-C(113)-Cl(1A)	111.5(13)
Cl(2A)-C(113)-H(11I)	109.3
Cl(1A)-C(113)-H(11I)	109.3
Cl(2A)-C(113)-H(11J)	109.3
Cl(1A)-C(113)-H(11J)	109.3
H(11I)-C(113)-H(11J)	108.0
Cl(2B)-C(114)-Cl(1B)	108(2)
Cl(2B)-C(114)-H(11K)	110.1
Cl(1B)-C(114)-H(11K)	110.1

Cl(2B)-C(114)-H(11L)	110.1
Cl(1B)-C(114)-H(11L)	110.1
H(11K)-C(114)-H(11L)	108.4
Cl(4A)-C(115)-Cl(3A)	123(2)
Cl(4A)-C(115)-H(11M)	106.7
Cl(3A)-C(115)-H(11M)	106.7
Cl(4A)-C(115)-H(11N)	106.7
Cl(3A)-C(115)-H(11N)	106.7
H(11M)-C(115)-H(11N)	106.6
Cl(4B)-C(116)-Cl(3B)	115.2(9)
Cl(4B)-C(116)-H(11O)	108.5
Cl(3B)-C(116)-H(11O)	108.5
Cl(4B)-C(116)-H(11P)	108.5
Cl(3B)-C(116)-H(11P)	108.5
H(11O)-C(116)-H(11P)	107.5
Cl(6)-C(117)-Cl(5)	114.8(6)
Cl(6)-C(117)-H(11Q)	108.6
Cl(5)-C(117)-H(11Q)	108.6
Cl(6)-C(117)-H(11R)	108.6
Cl(5)-C(117)-H(11R)	108.6
H(11Q)-C(117)-H(11R)	107.5

Symmetry transformations used to generate equivalent atoms:

Table S5. Anisotropic displacement parameters (A^2 x 10^3) for BIPPD. The anisotropic displacement factor exponent takes the form: -2 pi^2 [h^2 a*^2 U11 + ... + 2 h k a* b* U12]

	U11	U22	U33	U23	U13	U12
O(1)	33(1)	49(2)	64(2)	10(1)	2(1)	-12(1)
O(2)	49(2)	50(2)	71(2)	21(2)	0(2)	-6(1)
O(3)	45(2)	32(1)	63(2)	2(1)	11(1)	12(1)
O(4)	58(2)	47(2)	68(2)	6(2)	25(2)	8(1)
O(5)	47(2)	35(2)	64(2)	5(1)	14(1)	12(1)
O(6)	56(2)	48(2)	67(2)	7(2)	26(2)	10(1)
O(7)	50(2)	50(2)	64(2)	-17(2)	1(2)	6(2)
O(8)	35(1)	45(2)	60(2)	-1(1)	6(1)	12(1)
N(1)	36(2)	40(2)	60(2)	11(2)	4(2)	-10(1)
N(2)	28(1)	33(2)	56(2)	1(1)	4(1)	-7(1)
N(3)	35(2)	44(2)	55(2)	6(2)	4(1)	-9(1)
N(4)	37(2)	38(2)	54(2)	4(2)	2(1)	-6(1)
N(5)	40(2)	36(2)	60(2)	1(2)	16(2)	12(1)

N(6)	32(2)	34(2)	56(2)	-2(1)	8(1)	8(1)
N(7)	48(2)	38(2)	57(2)	-2(2)	15(2)	7(2)
N(8)	62(2)	40(2)	64(2)	2(2)	8(2)	8(2)
N(9)	40(2)	36(2)	60(2)	5(2)	18(2)	12(1)
N(10)	33(2)	31(2)	54(2)	2(1)	9(1)	9(1)
N(11)	53(2)	36(2)	56(2)	3(2)	12(2)	10(2)
N(12)	44(2)	34(2)	55(2)	5(1)	12(2)	8(1)
N(13)	38(2)	35(2)	58(2)	-8(2)	4(2)	10(1)
N(14)	32(2)	29(2)	52(2)	-1(1)	3(1)	6(1)
N(15)	34(2)	44(2)	56(2)	-3(2)	0(1)	8(1)
N(16)	38(2)	53(2)	49(2)	-4(2)	6(2)	5(2)
C(1)	33(2)	37(2)	55(2)	6(2)	9(2)	-6(2)
C(2)	32(2)	33(2)	54(2)	3(2)	6(2)	-4(2)
C(3)	30(2)	32(2)	49(2)	2(2)	3(2)	-5(1)
C(4)	37(2)	42(2)	52(2)	5(2)	6(2)	-8(2)
C(5)	32(2)	35(2)	52(2)	4(2)	5(2)	-4(2)
C(6)	32(2)	33(2)	49(2)	-1(2)	6(2)	-4(1)
C(7)	34(2)	35(2)	49(2)	1(2)	4(2)	-1(2)
C(8)	39(2)	40(2)	67(3)	6(2)	4(2)	-8(2)
C(9)	47(2)	37(2)	56(2)	5(2)	9(2)	2(2)
C(10)	71(3)	44(2)	63(3)	13(2)	8(2)	7(2)
C(11)	71(3)	66(3)	57(3)	8(2)	6(2)	17(3)
C(12)	60(3)	93(4)	46(3)	3(3)	0(2)	14(3)
C(13)	43(2)	78(3)	48(2)	-1(2)	3(2)	-2(2)
C(14)	38(2)	46(2)	46(2)	1(2)	7(2)	2(2)
C(15)	31(2)	38(2)	51(2)	-3(2)	4(2)	-2(2)
C(16)	34(2)	33(2)	59(3)	1(2)	4(2)	-4(2)
C(17)	32(2)	43(2)	53(2)	-8(2)	4(2)	-4(2)
C(18)	39(2)	55(3)	62(3)	-9(2)	6(2)	-12(2)
C(19)	38(2)	79(4)	61(3)	-12(3)	-4(2)	-6(2)
C(20)	48(2)	80(4)	50(3)	-6(2)	-3(2)	3(2)
C(21)	47(2)	54(3)	52(3)	2(2)	4(2)	2(2)
C(22)	37(2)	45(2)	46(2)	-3(2)	6(2)	-4(2)
C(23)	46(2)	38(2)	72(3)	13(2)	7(2)	-14(2)
C(24)	46(2)	56(3)	70(3)	20(2)	2(2)	-10(2)
C(25)	47(3)	75(4)	81(4)	30(3)	6(2)	-13(2)
C(26)	59(3)	109(5)	88(4)	43(4)	15(3)	2(3)
C(27)	70(4)	150(8)	121(6)	60(6)	33(4)	-8(5)
C(28)	102(4)	208(8)	159(6)	24(7)	68(5)	6(6)
C(29)	34(2)	34(2)	49(2)	-4(2)	9(2)	4(2)
C(30)	37(2)	29(2)	50(2)	-1(2)	8(2)	7(2)
C(31)	32(2)	31(2)	53(2)	-4(2)	4(2)	5(1)
C(32)	45(2)	36(2)	48(2)	1(2)	11(2)	6(2)
C(33)	36(2)	33(2)	50(2)	0(2)	8(2)	5(2)

C(34)	37(2)	30(2)	51(2)	-2(2)	3(2)	5(2)
C(35)	36(2)	37(2)	52(2)	-3(2)	9(2)	3(2)
C(36)	45(2)	48(2)	71(3)	-6(2)	20(2)	7(2)
C(37)	54(3)	52(3)	61(3)	-10(2)	21(2)	-4(2)
C(38)	83(4)	76(4)	86(4)	-3(3)	48(3)	3(3)
C(39)	108(5)	88(5)	73(4)	-4(3)	48(4)	-16(4)
C(40)	112(5)	74(4)	59(3)	9(3)	20(3)	-18(4)
C(41)	86(4)	48(3)	59(3)	3(2)	11(3)	-2(2)
C(42)	52(2)	43(2)	50(2)	-4(2)	10(2)	-5(2)
C(43)	38(2)	35(2)	57(2)	-2(2)	-2(2)	6(2)
C(44)	42(2)	40(2)	87(4)	-9(2)	-2(2)	9(2)
C(45)	76(4)	35(2)	90(4)	-3(2)	-33(3)	9(2)
C(46)	117(4)	61(3)	95(4)	4(2)	-17(3)	3(3)
C(47)	146(8)	50(3)	105(6)	16(4)	-35(5)	22(4)
C(48)	212(12)	63(4)	92(6)	23(4)	-4(7)	3(6)
C(49)	117(4)	61(3)	95(4)	4(2)	-17(3)	3(3)
C(50)	102(4)	40(2)	57(3)	8(2)	-7(3)	-6(3)
C(51)	39(2)	42(2)	70(3)	-2(2)	21(2)	12(2)
C(52)	59(3)	41(2)	69(3)	0(2)	25(2)	8(2)
C(53)	72(3)	51(3)	95(4)	-6(3)	41(3)	8(2)
C(54)	118(6)	72(4)	103(5)	-25(4)	55(5)	-2(4)
C(55)	135(9)	142(9)	238(14)	-107(10)	85(9)	-8(7)
C(56)	221(17)	165(13)	300(20)	-132(15)	89(16)	4(12)
C(57)	37(2)	30(2)	54(2)	-2(2)	8(2)	5(2)
C(58)	34(2)	33(2)	49(2)	2(2)	7(2)	7(2)
C(59)	34(2)	32(2)	47(2)	2(2)	8(2)	5(2)
C(60)	38(2)	33(2)	51(2)	2(2)	13(2)	4(2)
C(61)	38(2)	33(2)	51(2)	2(2)	9(2)	6(2)
C(62)	35(2)	32(2)	46(2)	-1(2)	4(2)	5(2)
C(63)	38(2)	34(2)	47(2)	0(2)	6(2)	3(2)
C(64)	50(2)	41(2)	62(3)	5(2)	19(2)	9(2)
C(65)	49(2)	50(2)	51(2)	1(2)	10(2)	1(2)
C(66)	76(4)	72(3)	64(3)	7(3)	27(3)	1(3)
C(67)	104(5)	84(4)	61(3)	7(3)	35(3)	-17(4)
C(68)	119(5)	63(3)	60(3)	16(3)	17(3)	-16(4)
C(69)	101(4)	47(3)	60(3)	9(2)	10(3)	1(3)
C(70)	61(3)	39(2)	46(2)	-2(2)	3(2)	-1(2)
C(71)	36(2)	30(2)	50(2)	1(2)	5(2)	1(2)
C(72)	38(2)	33(2)	59(2)	1(2)	6(2)	6(2)
C(73)	42(2)	35(2)	53(2)	3(2)	-1(2)	2(2)
C(74)	50(2)	39(2)	64(3)	4(2)	-4(2)	11(2)
C(75)	64(3)	42(2)	65(3)	13(2)	-9(2)	3(2)
C(76)	72(3)	53(3)	49(3)	15(2)	-1(2)	-5(2)
C(77)	55(2)	52(2)	49(2)	7(2)	10(2)	0(2)

C(78)	45(2)	33(2)	47(2)	3(2)	0(2)	3(2)
C(79)	39(2)	44(2)	69(3)	4(2)	20(2)	13(2)
C(80)	52(2)	44(2)	61(3)	6(2)	19(2)	11(2)
C(81)	56(3)	49(2)	64(3)	5(2)	25(2)	13(2)
C(82)	75(3)	53(3)	70(3)	-3(2)	29(3)	3(2)
C(83)	91(4)	69(4)	95(5)	-11(3)	39(4)	16(3)
C(84)	164(9)	71(4)	115(6)	-17(4)	56(6)	21(5)
C(85)	38(2)	34(2)	52(2)	-3(2)	6(2)	4(2)
C(86)	35(2)	33(2)	46(2)	-2(2)	5(2)	5(2)
C(87)	32(2)	35(2)	45(2)	2(2)	7(2)	3(1)
C(88)	33(2)	33(2)	47(2)	-1(2)	8(2)	6(1)
C(89)	31(2)	30(2)	49(2)	2(2)	4(2)	6(1)
C(90)	33(2)	31(2)	49(2)	2(2)	5(2)	3(1)
C(91)	32(2)	35(2)	49(2)	4(2)	4(2)	3(2)
C(92)	36(2)	42(2)	59(3)	2(2)	3(2)	7(2)
C(93)	38(2)	47(2)	54(2)	10(2)	6(2)	7(2)
C(94)	45(2)	63(3)	70(3)	14(2)	3(2)	15(2)
C(95)	42(2)	86(4)	72(3)	13(3)	-6(2)	13(2)
C(96)	47(3)	100(4)	57(3)	2(3)	-8(2)	-3(3)
C(97)	53(3)	67(3)	59(3)	-5(2)	-2(2)	2(2)
C(98)	34(2)	56(3)	50(2)	7(2)	0(2)	2(2)
C(99)	34(2)	33(2)	51(2)	1(2)	5(2)	5(2)
C(100)	48(2)	41(2)	66(3)	-11(2)	3(2)	9(2)
C(101)	55(2)	44(2)	50(2)	-3(2)	2(2)	-1(2)
C(102)	79(4)	63(3)	65(3)	-15(3)	-2(3)	6(3)
C(103)	94(4)	81(4)	50(3)	-16(3)	-2(3)	-12(3)
C(104)	64(3)	114(5)	51(3)	-3(3)	-9(2)	-16(3)
C(105)	44(2)	101(4)	51(3)	-2(3)	3(2)	-2(3)
C(106)	45(2)	54(3)	45(2)	4(2)	3(2)	-3(2)
C(107)	45(2)	35(2)	69(3)	-9(2)	9(2)	10(2)
C(108)	48(2)	54(3)	60(3)	-13(2)	10(2)	7(2)
C(109)	54(3)	60(3)	78(3)	-25(3)	12(2)	7(2)
C(110)	73(4)	89(4)	89(4)	-26(4)	33(3)	-6(3)
C(111)	118(7)	109(6)	149(8)	-44(6)	71(6)	9(5)
C(112)	102(4)	208(8)	159(6)	24(7)	68(5)	6(6)
C(113)	172(7)	107(5)	128(5)	9(4)	62(5)	13(5)
Cl(1A)	130(3)	164(5)	157(5)	-47(3)	-14(3)	-21(3)
Cl(2A)	110(6)	126(3)	220(4)	-40(3)	-54(6)	-12(5)
C(114)	172(7)	107(5)	128(5)	9(4)	62(5)	13(5)
Cl(1B)	130(3)	164(5)	157(5)	-47(3)	-14(3)	-21(3)
Cl(2B)	110(6)	126(3)	220(4)	-40(3)	-54(6)	-12(5)
C(115)	172(7)	107(5)	128(5)	9(4)	62(5)	13(5)
Cl(3A)	165(4)	145(4)	223(6)	13(4)	-57(5)	9(3)
Cl(4A)	208(5)	241(6)	213(5)	-21(5)	98(4)	-12(5)

C(116)	172(7)	107(5)	128(5)	9(4)	62(5)	13(5)
Cl(3B)	165(4)	145(4)	223(6)	13(4)	-57(5)	9(3)
Cl(4B)	208(5)	241(6)	213(5)	-21(5)	98(4)	-12(5)
C(117)	172(7)	107(5)	128(5)	9(4)	62(5)	13(5)
Cl(5)	132(2)	204(4)	195(3)	-41(3)	-16(2)	3(2)
Cl(6)	123(2)	160(3)	202(3)	-1(2)	42(2)	-21(2)

Table S6. Hydrogen coordinates (x 10^4) and isotropic displacement parameters (A^2 x 10^3) for BIPPD.

	x	y	z	U(eq)
H(2A)	3313	6898	251	47
H(3A)	5357	7546	868	54
H(4A)	2670	8472	-917	52
H(6A)	1157	5917	5294	49
H(7A)	1925	3946	5863	56
H(8B)	2499	6422	4107	66
H(10M)	8753	2522	4696	47
H(11I)	8040	4495	4086	58
H(12B)	7341	2075	5854	52
H(14A)	6638	5405	4643	45
H(15A)	7294	6964	5811	54
H(16B)	4552	5982	4107	56
H(8A)	3931	6288	1005	58
H(10A)	4667	5559	1877	71
H(11A)	5659	5539	2388	78
H(12A)	6469	6292	2273	81
H(13A)	6313	7085	1633	68
H(16A)	2110	6829	-292	51
H(18A)	801	6814	-919	63
H(19A)	141	7421	-1523	72
H(20A)	517	8370	-1869	72
H(21A)	1573	8755	-1605	62
H(23A)	5113	9738	-63	63
H(23B)	4491	9923	-450	63
H(24A)	4883	9354	-1116	69
H(24B)	5463	9062	-735	69
H(25A)	5929	10129	-631	81
H(25B)	5364	10393	-1042	81
H(26A)	6275	9430	-1287	102
H(26B)	5742	9755	-1693	102
H(27A)	6804	10460	-1166	135

H(27B)	6294	10745	-1613	135
H(28A)	7271	10436	-1966	228
H(28B)	7195	9716	-1771	228
H(28C)	6686	10000	-2217	228
H(36A)	673	5364	6054	64
H(38A)	150	4737	6942	95
H(39A)	282	3841	7469	104
H(40A)	1034	3031	7342	97
H(41A)	1671	3102	6655	77
H(44A)	987	7038	4778	68
H(46A)	819	8268	4141	112
H(47A)	1288	8789	3465	126
H(48A)	2176	8393	3135	149
H(49A)	2639	7402	3415	112
H(51A)	3934	4087	4927	59
H(51B)	4062	4666	4553	59
H(52A)	3179	3633	4243	66
H(52B)	3384	4185	3872	66
H(53A)	4278	3240	4336	84
H(53B)	4452	3771	3936	84
H(54A)	3501	2777	3662	113
H(54B)	3753	3273	3268	113
H(55A)	4786	2782	3364	200
H(55B)	4579	2318	3799	200
H(56A)	4557	1765	3034	337
H(56B)	3843	1805	3196	337
H(56C)	4050	2269	2761	337
H(64A)	9240	3026	3934	60
H(66A)	9820	3603	3051	83
H(67A)	9789	4528	2546	97
H(68A)	9080	5370	2672	96
H(69A)	8395	5328	3315	83
H(72A)	8886	1405	5248	52
H(74A)	8988	189	5890	62
H(75A)	8474	-353	6501	70
H(76A)	7562	79	6828	70
H(77A)	7140	1091	6538	62
H(79A)	5844	3829	5354	60
H(79B)	5983	4390	4968	60
H(80A)	6691	4884	5669	62
H(80B)	6442	4359	6043	62
H(81A)	5590	5277	5486	66
H(81B)	5375	4780	5896	66
H(82A)	6270	5811	6150	77

H(82B)	6040	5321	6558	77
H(83A)	5191	6229	5998	99
H(83B)	4976	5750	6419	99
H(84A)	5124	6781	6760	170
H(84B)	5640	6305	7058	170
H(84C)	5856	6785	6637	170
H(92A)	7819	5305	5212	55
H(94A)	9122	5251	5857	72
H(95A)	9771	5835	6483	81
H(96A)	9414	6810	6814	83
H(97A)	8384	7223	6513	73
H(10B)	6045	4881	3829	63
H(10C)	5322	4233	2907	84
H(10D)	4303	4198	2418	91
H(10E)	3452	4834	2643	93
H(10F)	3595	5555	3337	79
H(10G)	4819	8204	5016	60
H(10H)	5452	8394	5390	60
H(10I)	4485	7554	5712	65
H(10J)	5072	7860	6077	65
H(10K)	4601	8891	5980	76
H(10L)	4037	8622	5571	76
H(11B)	4198	8304	6639	98
H(11C)	3665	7972	6238	98
H(11D)	3660	9307	6541	145
H(11E)	3170	9038	6078	145
H(11F)	2656	9101	6817	228
H(11G)	2695	8367	6647	228
H(11H)	3186	8635	7110	228
H(11I)	9014	1750	2103	159
H(11J)	9005	1306	2598	159
H(11K)	8993	2255	2266	159
H(11L)	9278	1549	2393	159
H(11M)	6787	5180	2053	159
H(11N)	7409	5552	2305	159
H(11O)	7640	4292	1856	159
H(11P)	8006	4079	2396	159
H(11Q)	6577	1704	7971	159
H(11R)	6281	2339	7696	159

Table S7. Torsion angles [deg] for BIPPD.

C(4)-N(1)-C(1)-O(1)	178.6(4)
C(23)-N(1)-C(1)-O(1)	5.0(7)
C(4)-N(1)-C(1)-C(2)	-1.3(5)
C(23)-N(1)-C(1)-C(2)	-174.8(4)
O(1)-C(1)-C(2)-C(3)	6.2(10)
N(1)-C(1)-C(2)-C(3)	-174.0(6)
O(1)-C(1)-C(2)-C(5)	-178.2(5)
N(1)-C(1)-C(2)-C(5)	1.7(5)
C(6)-N(2)-C(3)-C(2)	-0.5(5)
C(6)-N(2)-C(3)-C(7)	177.7(4)
C(5)-C(2)-C(3)-N(2)	0.6(5)
C(1)-C(2)-C(3)-N(2)	176.2(6)
C(5)-C(2)-C(3)-C(7)	-177.5(4)
C(1)-C(2)-C(3)-C(7)	-1.8(10)
C(1)-N(1)-C(4)-O(2)	179.7(4)
C(23)-N(1)-C(4)-O(2)	-6.7(7)
C(1)-N(1)-C(4)-C(5)	0.4(5)
C(23)-N(1)-C(4)-C(5)	174.0(4)
C(3)-C(2)-C(5)-C(6)	-0.4(5)
C(1)-C(2)-C(5)-C(6)	-177.7(4)
C(3)-C(2)-C(5)-C(4)	175.7(4)
C(1)-C(2)-C(5)-C(4)	-1.5(5)
O(2)-C(4)-C(5)-C(6)	-4.5(10)
N(1)-C(4)-C(5)-C(6)	174.7(6)
O(2)-C(4)-C(5)-C(2)	-178.5(5)
N(1)-C(4)-C(5)-C(2)	0.7(5)
C(3)-N(2)-C(6)-C(5)	0.3(5)
C(3)-N(2)-C(6)-C(15)	179.3(4)
C(2)-C(5)-C(6)-N(2)	0.1(5)
C(4)-C(5)-C(6)-N(2)	-174.0(6)
C(2)-C(5)-C(6)-C(15)	-178.9(4)
C(4)-C(5)-C(6)-C(15)	7.1(9)
C(14)-N(3)-C(7)-C(8)	-0.5(5)
C(14)-N(3)-C(7)-C(3)	176.8(4)
N(2)-C(3)-C(7)-N(3)	177.3(4)
C(2)-C(3)-C(7)-N(3)	-5.0(7)
N(2)-C(3)-C(7)-C(8)	-6.1(7)
C(2)-C(3)-C(7)-C(8)	171.6(5)
N(3)-C(7)-C(8)-C(9)	0.6(5)
C(3)-C(7)-C(8)-C(9)	-176.3(4)

C(7)-C(8)-C(9)-C(10)	179.4(5)
C(7)-C(8)-C(9)-C(14)	-0.5(5)
C(14)-C(9)-C(10)-C(11)	-0.2(7)
C(8)-C(9)-C(10)-C(11)	180.0(5)
C(9)-C(10)-C(11)-C(12)	-0.3(8)
C(10)-C(11)-C(12)-C(13)	-0.3(9)
C(11)-C(12)-C(13)-C(14)	1.3(8)
C(7)-N(3)-C(14)-C(13)	179.1(5)
C(7)-N(3)-C(14)-C(9)	0.2(5)
C(12)-C(13)-C(14)-N(3)	179.4(5)
C(12)-C(13)-C(14)-C(9)	-1.8(7)
C(10)-C(9)-C(14)-N(3)	-179.7(4)
C(8)-C(9)-C(14)-N(3)	0.2(5)
C(10)-C(9)-C(14)-C(13)	1.3(7)
C(8)-C(9)-C(14)-C(13)	-178.8(4)
C(22)-N(4)-C(15)-C(16)	0.2(5)
C(22)-N(4)-C(15)-C(6)	-179.8(4)
N(2)-C(6)-C(15)-N(4)	-177.8(4)
C(5)-C(6)-C(15)-N(4)	1.0(6)
N(2)-C(6)-C(15)-C(16)	2.1(7)
C(5)-C(6)-C(15)-C(16)	-179.1(5)
N(4)-C(15)-C(16)-C(17)	-0.4(5)
C(6)-C(15)-C(16)-C(17)	179.6(4)
C(15)-C(16)-C(17)-C(18)	-177.2(5)
C(15)-C(16)-C(17)-C(22)	0.5(5)
C(22)-C(17)-C(18)-C(19)	-1.2(7)
C(16)-C(17)-C(18)-C(19)	176.4(5)
C(17)-C(18)-C(19)-C(20)	1.3(8)
C(18)-C(19)-C(20)-C(21)	-0.6(8)
C(19)-C(20)-C(21)-C(22)	-0.2(8)
C(15)-N(4)-C(22)-C(21)	177.2(4)
C(15)-N(4)-C(22)-C(17)	0.1(5)
C(20)-C(21)-C(22)-N(4)	-176.5(5)
C(20)-C(21)-C(22)-C(17)	0.2(7)
C(18)-C(17)-C(22)-N(4)	177.8(4)
C(16)-C(17)-C(22)-N(4)	-0.4(5)
C(18)-C(17)-C(22)-C(21)	0.5(7)
C(16)-C(17)-C(22)-C(21)	-177.7(4)
C(1)-N(1)-C(23)-C(24)	86.4(6)
C(4)-N(1)-C(23)-C(24)	-86.5(5)
N(1)-C(23)-C(24)-C(25)	-171.4(4)
C(23)-C(24)-C(25)-C(26)	176.2(5)
C(24)-C(25)-C(26)-C(27)	-174.8(6)
C(25)-C(26)-C(27)-C(28)	174.7(7)

C(32)-N(5)-C(29)-O(3)	178.1(4)
C(51)-N(5)-C(29)-O(3)	2.5(7)
C(32)-N(5)-C(29)-C(30)	-1.1(5)
C(51)-N(5)-C(29)-C(30)	-176.7(4)
O(3)-C(29)-C(30)-C(31)	7.1(9)
N(5)-C(29)-C(30)-C(31)	-173.7(6)
O(3)-C(29)-C(30)-C(33)	-177.6(4)
N(5)-C(29)-C(30)-C(33)	1.5(5)
C(34)-N(6)-C(31)-C(30)	-0.6(5)
C(34)-N(6)-C(31)-C(35)	176.9(4)
C(33)-C(30)-C(31)-N(6)	1.0(5)
C(29)-C(30)-C(31)-N(6)	176.2(5)
C(33)-C(30)-C(31)-C(35)	-176.4(4)
C(29)-C(30)-C(31)-C(35)	-1.1(9)
C(29)-N(5)-C(32)-O(4)	179.0(4)
C(51)-N(5)-C(32)-O(4)	-5.4(7)
C(29)-N(5)-C(32)-C(33)	0.3(5)
C(51)-N(5)-C(32)-C(33)	175.9(4)
C(31)-C(30)-C(33)-C(34)	-0.9(5)
C(29)-C(30)-C(33)-C(34)	-178.0(4)
C(31)-C(30)-C(33)-C(32)	175.6(4)
C(29)-C(30)-C(33)-C(32)	-1.4(5)
O(4)-C(32)-C(33)-C(34)	-3.1(10)
N(5)-C(32)-C(33)-C(34)	175.4(6)
O(4)-C(32)-C(33)-C(30)	-177.8(5)
N(5)-C(32)-C(33)-C(30)	0.7(5)
C(31)-N(6)-C(34)-C(33)	0.1(5)
C(31)-N(6)-C(34)-C(43)	178.2(4)
C(30)-C(33)-C(34)-N(6)	0.5(5)
C(32)-C(33)-C(34)-N(6)	-174.2(6)
C(30)-C(33)-C(34)-C(43)	-177.5(4)
C(32)-C(33)-C(34)-C(43)	7.8(9)
C(42)-N(7)-C(35)-C(36)	-1.7(5)
C(42)-N(7)-C(35)-C(31)	174.5(4)
N(6)-C(31)-C(35)-N(7)	179.0(4)
C(30)-C(31)-C(35)-N(7)	-4.1(7)
N(6)-C(31)-C(35)-C(36)	-5.8(7)
C(30)-C(31)-C(35)-C(36)	171.1(5)
N(7)-C(35)-C(36)-C(37)	1.8(5)
C(31)-C(35)-C(36)-C(37)	-173.8(4)
C(35)-C(36)-C(37)-C(38)	179.1(6)
C(35)-C(36)-C(37)-C(42)	-1.3(6)
C(42)-C(37)-C(38)-C(39)	1.8(9)
C(36)-C(37)-C(38)-C(39)	-178.7(7)

C(37)-C(38)-C(39)-C(40)	-1.2(12)
C(38)-C(39)-C(40)-C(41)	-0.7(12)
C(39)-C(40)-C(41)-C(42)	1.9(10)
C(35)-N(7)-C(42)-C(41)	-178.7(5)
C(35)-N(7)-C(42)-C(37)	0.9(5)
C(40)-C(41)-C(42)-N(7)	178.2(5)
C(40)-C(41)-C(42)-C(37)	-1.3(8)
C(38)-C(37)-C(42)-N(7)	179.9(5)
C(36)-C(37)-C(42)-N(7)	0.3(5)
C(38)-C(37)-C(42)-C(41)	-0.5(8)
C(36)-C(37)-C(42)-C(41)	179.8(5)
C(50)-N(8)-C(43)-C(44)	0.5(6)
C(50)-N(8)-C(43)-C(34)	178.4(4)
N(6)-C(34)-C(43)-N(8)	-175.5(4)
C(33)-C(34)-C(43)-N(8)	2.2(7)
N(6)-C(34)-C(43)-C(44)	1.9(8)
C(33)-C(34)-C(43)-C(44)	179.6(5)
N(8)-C(43)-C(44)-C(45)	-0.6(5)
C(34)-C(43)-C(44)-C(45)	-178.2(5)
C(43)-C(44)-C(45)-C(50)	0.6(6)
C(43)-C(44)-C(45)-C(46)	-178.4(7)
C(44)-C(45)-C(46)-C(47)	179.4(6)
C(50)-C(45)-C(46)-C(47)	0.5(9)
C(45)-C(46)-C(47)-C(48)	-2.1(12)
C(46)-C(47)-C(48)-C(49)	2.0(15)
C(47)-C(48)-C(49)-C(50)	-0.4(13)
C(43)-N(8)-C(50)-C(49)	177.7(6)
C(43)-N(8)-C(50)-C(45)	-0.1(6)
C(48)-C(49)-C(50)-N(8)	-178.5(7)
C(48)-C(49)-C(50)-C(45)	-1.1(10)
C(44)-C(45)-C(50)-N(8)	-0.3(6)
C(46)-C(45)-C(50)-N(8)	178.9(5)
C(44)-C(45)-C(50)-C(49)	-178.2(6)
C(46)-C(45)-C(50)-C(49)	1.0(9)
C(29)-N(5)-C(51)-C(52)	87.7(5)
C(32)-N(5)-C(51)-C(52)	-87.5(5)
N(5)-C(51)-C(52)-C(53)	-173.9(4)
C(51)-C(52)-C(53)-C(54)	176.3(5)
C(52)-C(53)-C(54)-C(55)	-173.6(8)
C(53)-C(54)-C(55)-C(56)	174.6(12)
C(60)-N(9)-C(57)-O(5)	-178.9(4)
C(79)-N(9)-C(57)-O(5)	0.9(7)
C(60)-N(9)-C(57)-C(58)	0.1(5)
C(79)-N(9)-C(57)-C(58)	179.9(4)

O(5)-C(57)-C(58)-C(59)	-6.3(10)
N(9)-C(57)-C(58)-C(59)	174.8(6)
O(5)-C(57)-C(58)-C(61)	178.1(4)
N(9)-C(57)-C(58)-C(61)	-0.8(5)
C(61)-C(58)-C(59)-N(10)	-0.5(5)
C(57)-C(58)-C(59)-N(10)	-176.2(6)
C(61)-C(58)-C(59)-C(63)	176.1(4)
C(57)-C(58)-C(59)-C(63)	0.5(10)
C(62)-N(10)-C(59)-C(58)	0.4(5)
C(62)-N(10)-C(59)-C(63)	-176.6(4)
C(57)-N(9)-C(60)-O(6)	-178.5(4)
C(79)-N(9)-C(60)-O(6)	1.7(7)
C(57)-N(9)-C(60)-C(61)	0.7(5)
C(79)-N(9)-C(60)-C(61)	-179.1(4)
C(59)-C(58)-C(61)-C(62)	0.5(5)
C(57)-C(58)-C(61)-C(62)	177.7(4)
C(59)-C(58)-C(61)-C(60)	-176.0(4)
C(57)-C(58)-C(61)-C(60)	1.2(5)
O(6)-C(60)-C(61)-C(62)	3.4(10)
N(9)-C(60)-C(61)-C(62)	-175.6(6)
O(6)-C(60)-C(61)-C(58)	177.9(5)
N(9)-C(60)-C(61)-C(58)	-1.1(5)
C(58)-C(61)-C(62)-N(10)	-0.2(5)
C(60)-C(61)-C(62)-N(10)	174.3(6)
C(58)-C(61)-C(62)-C(71)	177.7(4)
C(60)-C(61)-C(62)-C(71)	-7.8(9)
C(59)-N(10)-C(62)-C(61)	-0.2(5)
C(59)-N(10)-C(62)-C(71)	-178.1(4)
C(70)-N(11)-C(63)-C(64)	0.8(5)
C(70)-N(11)-C(63)-C(59)	-177.0(4)
C(58)-C(59)-C(63)-C(64)	-170.7(5)
N(10)-C(59)-C(63)-C(64)	5.5(7)
C(58)-C(59)-C(63)-N(11)	6.5(7)
N(10)-C(59)-C(63)-N(11)	-177.3(4)
N(11)-C(63)-C(64)-C(65)	-0.7(5)
C(59)-C(63)-C(64)-C(65)	176.7(4)
C(63)-C(64)-C(65)-C(70)	0.4(6)
C(63)-C(64)-C(65)-C(66)	-177.6(6)
C(70)-C(65)-C(66)-C(67)	2.0(9)
C(64)-C(65)-C(66)-C(67)	179.8(6)
C(65)-C(66)-C(67)-C(68)	-0.9(10)
C(66)-C(67)-C(68)-C(69)	0.3(11)
C(67)-C(68)-C(69)-C(70)	-0.9(10)
C(63)-N(11)-C(70)-C(69)	-179.3(5)

C(63)-N(11)-C(70)-C(65)	-0.5(5)
C(68)-C(69)-C(70)-N(11)	-179.3(6)
C(68)-C(69)-C(70)-C(65)	2.0(8)
C(66)-C(65)-C(70)-N(11)	178.4(5)
C(64)-C(65)-C(70)-N(11)	0.1(5)
C(66)-C(65)-C(70)-C(69)	-2.6(8)
C(64)-C(65)-C(70)-C(69)	179.0(5)
C(78)-N(12)-C(71)-C(72)	0.5(5)
C(78)-N(12)-C(71)-C(62)	179.8(4)
C(61)-C(62)-C(71)-C(72)	179.8(5)
N(10)-C(62)-C(71)-C(72)	-2.8(7)
C(61)-C(62)-C(71)-N(12)	0.7(7)
N(10)-C(62)-C(71)-N(12)	178.2(4)
N(12)-C(71)-C(72)-C(73)	-0.4(5)
C(62)-C(71)-C(72)-C(73)	-179.5(4)
C(71)-C(72)-C(73)-C(74)	177.7(5)
C(71)-C(72)-C(73)-C(78)	0.2(5)
C(78)-C(73)-C(74)-C(75)	0.4(7)
C(72)-C(73)-C(74)-C(75)	-176.8(5)
C(73)-C(74)-C(75)-C(76)	0.0(8)
C(74)-C(75)-C(76)-C(77)	-0.1(8)
C(75)-C(76)-C(77)-C(78)	-0.3(7)
C(71)-N(12)-C(78)-C(77)	-177.1(4)
C(71)-N(12)-C(78)-C(73)	-0.4(5)
C(76)-C(77)-C(78)-N(12)	177.2(5)
C(76)-C(77)-C(78)-C(73)	0.8(7)
C(74)-C(73)-C(78)-N(12)	-177.9(4)
C(72)-C(73)-C(78)-N(12)	0.1(5)
C(74)-C(73)-C(78)-C(77)	-0.9(7)
C(72)-C(73)-C(78)-C(77)	177.1(4)
C(57)-N(9)-C(79)-C(80)	-89.9(5)
C(60)-N(9)-C(79)-C(80)	89.9(5)
N(9)-C(79)-C(80)-C(81)	171.4(4)
C(79)-C(80)-C(81)-C(82)	-175.4(4)
C(80)-C(81)-C(82)-C(83)	178.8(5)
C(81)-C(82)-C(83)-C(84)	-178.2(6)
C(88)-N(13)-C(85)-O(7)	179.1(4)
C(107)-N(13)-C(85)-O(7)	-4.9(7)
C(88)-N(13)-C(85)-C(86)	0.8(5)
C(107)-N(13)-C(85)-C(86)	176.9(4)
O(7)-C(85)-C(86)-C(87)	-2.5(10)
N(13)-C(85)-C(86)-C(87)	175.5(5)
O(7)-C(85)-C(86)-C(89)	-177.4(5)
N(13)-C(85)-C(86)-C(89)	0.7(5)

C(89)-C(86)-C(87)-N(14)	0.7(4)
C(85)-C(86)-C(87)-N(14)	-174.1(5)
C(89)-C(86)-C(87)-C(91)	-177.0(4)
C(85)-C(86)-C(87)-C(91)	8.2(9)
C(90)-N(14)-C(87)-C(86)	-0.5(4)
C(90)-N(14)-C(87)-C(91)	177.4(4)
C(85)-N(13)-C(88)-O(8)	176.4(4)
C(107)-N(13)-C(88)-O(8)	0.4(7)
C(85)-N(13)-C(88)-C(89)	-1.9(5)
C(107)-N(13)-C(88)-C(89)	-177.9(4)
C(87)-C(86)-C(89)-C(90)	-0.7(5)
C(85)-C(86)-C(89)-C(90)	175.8(4)
C(87)-C(86)-C(89)-C(88)	-178.4(3)
C(85)-C(86)-C(89)-C(88)	-1.9(5)
O(8)-C(88)-C(89)-C(90)	7.8(9)
N(13)-C(88)-C(89)-C(90)	-174.0(6)
O(8)-C(88)-C(89)-C(86)	-175.9(4)
N(13)-C(88)-C(89)-C(86)	2.3(4)
C(86)-C(89)-C(90)-N(14)	0.4(4)
C(88)-C(89)-C(90)-N(14)	176.7(5)
C(86)-C(89)-C(90)-C(99)	-175.2(4)
C(88)-C(89)-C(90)-C(99)	1.0(9)
C(87)-N(14)-C(90)-C(89)	0.0(4)
C(87)-N(14)-C(90)-C(99)	176.2(4)
C(98)-N(15)-C(91)-C(92)	0.0(5)
C(98)-N(15)-C(91)-C(87)	178.8(4)
C(86)-C(87)-C(91)-C(92)	176.3(5)
N(14)-C(87)-C(91)-C(92)	-1.0(7)
C(86)-C(87)-C(91)-N(15)	-2.1(6)
N(14)-C(87)-C(91)-N(15)	-179.5(4)
N(15)-C(91)-C(92)-C(93)	-0.3(5)
C(87)-C(91)-C(92)-C(93)	-178.8(4)
C(91)-C(92)-C(93)-C(94)	-179.3(5)
C(91)-C(92)-C(93)-C(98)	0.5(5)
C(98)-C(93)-C(94)-C(95)	-0.8(7)
C(92)-C(93)-C(94)-C(95)	179.0(5)
C(93)-C(94)-C(95)-C(96)	0.5(9)
C(94)-C(95)-C(96)-C(97)	-0.1(9)
C(95)-C(96)-C(97)-C(98)	-0.1(9)
C(91)-N(15)-C(98)-C(97)	178.9(5)
C(91)-N(15)-C(98)-C(93)	0.3(5)
C(96)-C(97)-C(98)-N(15)	-178.6(5)
C(96)-C(97)-C(98)-C(93)	-0.2(8)
C(94)-C(93)-C(98)-N(15)	179.3(4)

C(92)-C(93)-C(98)-N(15)	-0.5(5)
C(94)-C(93)-C(98)-C(97)	0.6(7)
C(92)-C(93)-C(98)-C(97)	-179.2(5)
C(106)-N(16)-C(99)-C(100)	0.1(5)
C(106)-N(16)-C(99)-C(90)	175.4(4)
C(89)-C(90)-C(99)-C(100)	163.4(5)
N(14)-C(90)-C(99)-C(100)	-11.6(7)
C(89)-C(90)-C(99)-N(16)	-10.8(7)
N(14)-C(90)-C(99)-N(16)	174.2(4)
N(16)-C(99)-C(100)-C(101)	0.8(5)
C(90)-C(99)-C(100)-C(101)	-174.0(4)
C(99)-C(100)-C(101)-C(106)	-1.3(5)
C(99)-C(100)-C(101)-C(102)	178.0(6)
C(106)-C(101)-C(102)-C(103)	-0.6(8)
C(100)-C(101)-C(102)-C(103)	-179.8(6)
C(101)-C(102)-C(103)-C(104)	-0.6(10)
C(102)-C(103)-C(104)-C(105)	1.4(11)
C(103)-C(104)-C(105)-C(106)	-1.0(10)
C(99)-N(16)-C(106)-C(105)	-179.9(5)
C(99)-N(16)-C(106)-C(101)	-0.9(5)
C(104)-C(105)-C(106)-N(16)	178.6(5)
C(104)-C(105)-C(106)-C(101)	-0.2(8)
C(102)-C(101)-C(106)-N(16)	-178.1(5)
C(100)-C(101)-C(106)-N(16)	1.3(5)
C(102)-C(101)-C(106)-C(105)	1.0(8)
C(100)-C(101)-C(106)-C(105)	-179.6(5)
C(88)-N(13)-C(107)-C(108)	88.0(5)
C(85)-N(13)-C(107)-C(108)	-87.6(5)
N(13)-C(107)-C(108)-C(109)	-170.7(4)
C(107)-C(108)-C(109)-C(110)	174.5(5)
C(108)-C(109)-C(110)-C(111)	-174.7(6)
C(109)-C(110)-C(111)-C(112)	171.5(9)

Symmetry transformations used to generate equivalent atoms:

Table S8. Hydrogen bonds for BIPPD [A and deg.].

D-H...A	d(D-H)	d(H...A)	d(D...A)	<(DHA)
N(2)-H(2A)...O(3)#1	0.86	1.99	2.847(4)	174.7
N(3)-H(3A)...O(1)	0.86	2.43	3.187(5)	147.5
N(4)-H(4A)...O(2)	0.86	2.18	2.975(5)	153.4
N(6)-H(6A)...O(1)#2	0.86	2.04	2.898(4)	171.6
N(7)-H(7A)...O(3)	0.86	2.38	3.140(5)	147.9
N(8)-H(8B)...O(4)	0.86	2.23	3.023(5)	152.8
N(10)-H(10M)...O(8)#3	0.86	2.05	2.901(4)	173.6
N(11)-H(11I)...O(5)	0.86	2.40	3.157(5)	147.5
N(12)-H(12B)...O(6)	0.86	2.19	2.985(5)	153.9
N(14)-H(14A)...O(5)	0.86	2.02	2.873(4)	169.6
N(15)-H(15A)...O(7)	0.86	2.18	2.982(5)	155.1
N(16)-H(16B)...O(8)	0.86	2.46	3.200(5)	144.8
C(13)-H(13A)...O(6)#1	0.93	2.64	3.147(6)	114.8
C(16)-H(16A)...O(3)#1	0.93	2.62	3.415(5)	144.4
C(44)-H(44A)...O(1)#2	0.93	2.53	3.349(7)	146.5
C(49)-H(49A)...Cl(1B)#4	0.93	2.86	3.784(16)	171.5
C(69)-H(69A)...O(2)#5	0.93	2.65	3.186(7)	117.3
C(92)-H(92A)...O(5)	0.93	2.61	3.421(6)	145.5
C(105)-H(10F)...O(4)	0.93	2.63	3.231(6)	123.0

Symmetry transformations used to generate equivalent atoms:
#1 x,-y+1,z-1/2 #2 x-1/2,-y+3/2,z+1/2 #3 x+1/2,y-1/2,z
#4 x-1/2,y+1/2,z #5 x+1/2,-y+3/2,z+1/2

6. Equal K/ Dimerization

$$K = 2.55 \times 10^{6}\ M^{-1} \pm 3\%$$

http://app.supramolecular.org/bindfit/view/16c094f4-1c8c-4f70-859a-a5e4f9f996ea

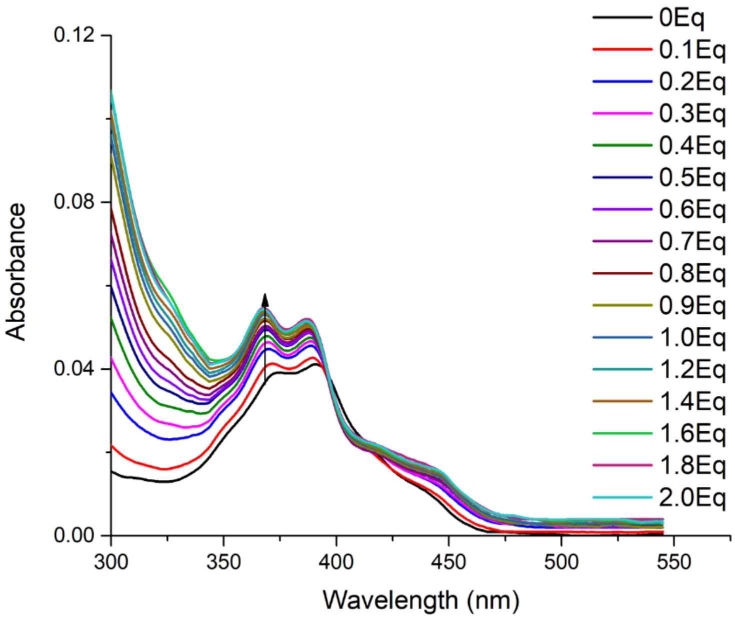

Figure S15. UV-visible binding studies of BIPPD in addition of TBA-H_2PO_4 in chlorobenzene collected at 298 K. Experiments were performed with the same concentration of complex BIPPD.

7. Computational Details

Energy Minimization Output:

SCF total energy: -2085.0547952 hartrees

Cartesian Coordinates (Angstroms)

Atom		X	Y	Z
1 N	N1	3.1796248	0.0044593	-0.9698025
2 C	C1	2.4432477	1.1839964	-0.8517044
3 C	C2	2.4053403	-1.1550844	-0.8735672
4 O	O1	2.9235464	2.2730021	-0.9095640
5 O	O2	2.8516628	-2.2551150	-0.9679451
6 C	C3	1.0584510	0.7391698	-0.6606267
7 C	C4	1.0359491	-0.6703249	-0.6642120
8 C	C5	-0.2149818	1.1870147	-0.4553187
9 C	C6	-0.2517374	-1.0795387	-0.4576861
10 N	N2	-0.9926004	0.0651510	-0.3373979

11 H	H5	-1.9975194	0.0988116	-0.2435785
12 C	C7	4.6138193	-0.0167621	-1.1828375
13 H	H2	4.8681193	0.8647480	-1.7577640
14 H	H1	4.8389477	-0.8918216	-1.7793663
15 C	C8	-0.8097817	-2.4246907	-0.3711514
16 C	C9	-0.7420568	2.5427231	-0.3580290
17 N	N3	-2.0514980	-2.6962788	0.1469779
18 H	H9	-2.6979047	-2.0760055	0.6025132
19 N	N4	-2.0688518	2.7984384	-0.1248327
20 H	H8	-2.7946573	2.1051806	-0.0454009
21 C	C10	-0.2140265	-3.5804744	-0.7741638
22 H	H4	0.7604918	-3.6573861	-1.2080354
23 C	C11	-1.1345619	-4.6400591	-0.4893124
24 C	C12	-3.3199117	-6.1447181	0.3208367
25 C	C13	-2.2621517	-4.0406486	0.0872134
26 C	C14	-1.1126225	-6.0308679	-0.6648770
27 C	C15	-2.1982943	-6.7683559	-0.2611920
28 C	C16	-3.3698269	-4.7843132	0.5028191
29 H	H12	-0.2569212	-6.5114848	-1.1070565
30 H	H13	-2.1977884	-7.8372105	-0.3877397
31 H	H14	-4.2190411	-4.2953157	0.9437393
32 H	H15	-4.1554988	-6.7498999	0.6277251
33 C	C17	-0.0533240	3.7127030	-0.4623396
34 H	H16	0.9982510	3.8015155	-0.6369571
35 C	C18	-1.0092933	4.7650455	-0.2863654
36 C	C19	-3.3145743	6.2540551	0.1298019
37 C	C20	-0.9329207	6.1639540	-0.2829702
38 C	C21	-2.2515788	4.1458450	-0.0793195
39 C	C22	-3.4182816	4.8837502	0.1328574
40 C	C23	-2.0783821	6.8944843	-0.0774043
41 H	H3	0.0106093	6.6578512	-0.4391705
42 H	H18	-4.3602602	4.3921698	0.2981683
43 H	H19	-2.0355751	7.9697694	-0.0719079
44 H	H20	-4.1951395	6.8513552	0.2917744
45 P	P1	-4.7398193	-0.1551105	0.7909925
46 O	O3	-3.7951942	0.6003987	-0.0754177
47 O	O4	-5.2450056	0.8485634	1.9398774
48 O	O5	-6.0687298	-0.4228914	-0.0678416
49 O	O6	-4.3008307	-1.4150733	1.4125667
50 H	H7	-5.4392030	0.3898467	2.7389238
51 H	H11	-6.1356872	0.1637871	-0.8011232
52 C	C24	5.4085354	-0.0462454	0.1211896
53 H	H10	5.1138697	-0.9235775	0.6916908
54 H	H17	5.1430318	0.8260486	0.7133958

55 C	C25	6.9170735	-0.0683206	-0.1208972
56 H	H6	7.1729841	-0.9381105	-0.7256907
57 H	H22	7.2019280	0.8068068	-0.7047426
58 C	C26	7.7332977	-0.0973605	1.1712539
59 H	H23	7.4487175	-0.9715372	1.7558312
60 H	H24	7.4774464	0.7712944	1.7770119
61 C	C27	9.2437051	-0.1194296	0.9364698
62 H	H21	9.5010011	-0.9881166	0.3322978
63 H	H26	9.5297340	0.7541542	0.3525845
64 C	C28	10.0532992	-0.1479082	2.2319760
65 H	H25	11.1211474	-0.1645230	2.0299792
66 H	H27	9.8142423	-1.0279184	2.8241274
67	H28	9.8451912	0.7269269	2.8432039

References

[1] S. K. Pollack, Y. M. Hijji, B. Kgobane, *Macromolecules* **1997**, *30*, 6709-6711.

[2] J. T. Brewster, H. Zafar, M. McVeigh, C. D. Wight, G. Anguera, A. Steinbrück, V. M. Lynch, J. L. Sessler, *J. Org. Chem.,* **2018**, *83*, 9568-9570.
